# Celloscope: a probabilistic model for marker-gene-driven cell type deconvolution in spatial transcriptomics data

**DOI:** 10.1186/s13059-023-02951-8

**Published:** 2023-05-17

**Authors:** Agnieszka Geras, Shadi Darvish Shafighi, Kacper Domżał, Igor Filipiuk, Alicja Rączkowska, Paulina Szymczak, Hosein Toosi, Leszek Kaczmarek, Łukasz Koperski, Jens Lagergren, Dominika Nowis, Ewa Szczurek

**Affiliations:** 1 Faculty of Mathematics and Information Science, Warsaw University of Technology, Warsaw, Poland; 2 Faculty of Mathematics, Informatics, and Mechanics, University of Warsaw, Warsaw, Poland; 3 Sorbonne Université, CNRS, IBPS, Laboratoire de Biologie Computationnelle et Quantitative - UMR, Paris, France; 4 KTH Royal Institute of Technology, Stockholm, Sweden; 5 BRAINCITY, Nencki Institute of Experimental Biology of the Polish Academy of Sciences, Warsaw, Poland; 6 Department of Pathology, Medical University of Warsaw, Warsaw, Poland; 7 Laboratory of Experimental Medicine, Medical University of Warsaw, Warsaw, Poland

**Keywords:** Probabilistic model, MCMC sampling, Spatial transcriptomics data, Cell types

## Abstract

Spatial transcriptomics maps gene expression across tissues, posing the challenge of determining the spatial arrangement of different cell types. However, spatial transcriptomics spots contain multiple cells. Therefore, the observed signal comes from mixtures of cells of different types. Here, we propose an innovative probabilistic model, Celloscope, that utilizes established prior knowledge on marker genes for cell type deconvolution from spatial transcriptomics data. Celloscope outperforms other methods on simulated data, successfully indicates known brain structures and spatially distinguishes between inhibitory and excitatory neuron types based in mouse brain tissue, and dissects large heterogeneity of immune infiltrate composition in prostate gland tissue.

**Supplementary Information:**

The online version contains supplementary material available at 10.1186/s13059-023-02951-8.

## Background

Gene expression is crucial for characterizing tissue functionality in both normal and abnormal conditions. For this reason, various high-throughput sequencing technologies measuring gene expression were developed. In contrast to standard bulk and single cell RNA sequencing (scRNA-seq) technologies [1–4], the groundbreaking spatial transcriptomics (ST) technology [5] enables to investigate the tissue functionality in an unprecedented, ultra-high, spatial resolution. ST captures RNA-sequencing measurements in multiple distinct spots that differ in resolution depending on the tissue and technology from subcellular, through 1–10, up to 100 cells per spot [6]. Importantly, for ST data characterized by a higher number of cells per spot, cell type mixtures decomposition is a necessity. Consequently, a number of computational methods for deconvolution of cell types in ST spots were proposed. All but one of these approaches rely on additional scRNA-seq measurements, ideally from the same tissue [7–16]. To perform the deconvolution, in the first step, the additional scRNA-seq data are used to compute gene expression profiles for the analyzed cell types. In the second step, the computed expression profiles are utilized to resolve the composition of cell types present at each ST spot. To this end, diverse statistical methods are applied, such as maximum a posteriori estimation in the case of Stereoscope [8] and maximum likelihood estimation in the case of RCTD [7]. Cell2location [11], as a Bayesian hierarchical framework, allows for introducing factorized priors. However, the choice of hyperparameters may greatly influence its performance [11]. BayesPrism [15] applies Markov chain Monte Carlo to jointly estimate gene expression profiles of cell types and states together with their proportions in ST spots. An alternative statistical strategy for deconvolution is to use log-normal [10] or weighted linear regression [13], utilizing the pre-computed gene expression levels for cell types as explanatory variables. Finally, Tangram [14] learns a mapping function that transfers scRNA-seq (or single nuclei RNA-seq) expression to the tissue space measured using ST, so that spatial correlation across the genes between the two techniques is maximized.

Integrating data obtained via two different experimental techniques such as scRNA-seq and ST may be prone to bias arising from cross-platform and batch effects, such as differences in the sequencing depth or only a partial overlap between sequenced cell types and genes. Moreover, such a reference data set measuring gene expression in cells of different types, ideally taken from exactly the same tissue sample, may not be accessible. This calls for methods designed for ST data that do not require an external reference in the form of scRNA-seq data. To this end, STdeconvolve [16] uses latent Dirichlet allocation (LDA) to simultaneously infer gene expression profiles and proportions of cell types in each ST spot. However, it does not rely on any external reference and the cell type decomposition is based solely on patterns of gene co-expression.

Hence, there is a dearth of methods for cell type deconvolution in ST data, which do not require a reference scRNA-seq dataset, but instead incorporate prior, domain knowledge that limits their hypothesis space and guides toward feasible solutions [17, 18].

To address these needs, here we present Celloscope―a novel method for decomposing cell type mixtures in ST spots, enabling to spatially map cell types in tissues examined with ST technology. Importantly, the innovative character of Celloscope involves using prior *qualitative* information on marker genes. Such marker genes are expected to be more highly expressed in their respective cell types compared to other types [19]. There is a large body of expert knowledge on marker genes for different cell types, established by multiple independent studies (as of 19.11.2022, PubMed search for “cell type marker genes” phrase returned 67,327 entries). Celloscope can leverage this wealth of knowledge and, as a consequence, is fully independent of scRNA-seq reference datasets. The expression level for each marker gene in each cell type is estimated as part of the model’s inference from ST data. Moreover, we estimate the admixture of additional, *a priori* unknown cell types.

The results of extensive experiments on simulated data demonstrated the excellent Celloscope’s performance. On top of that, Celloscope surpassed other methods that use scRNA-seq data as a reference to compute gene expression profiles in a simulation study. To further validate the correctness of the Celloscope’s assumptions and prove its usefulness, we applied it to data on the anterior section of the mouse brain. We also showed that Celloscope can be used to elucidate the cause of inflammation in a human prostate tissue [20]. These results indicate that Celloscope successfully leverages biological knowledge of cell type marker genes to accurately decompose cell type mixtures in ST data.

## Results

### Celloscope model overview

**Fig. 1:**
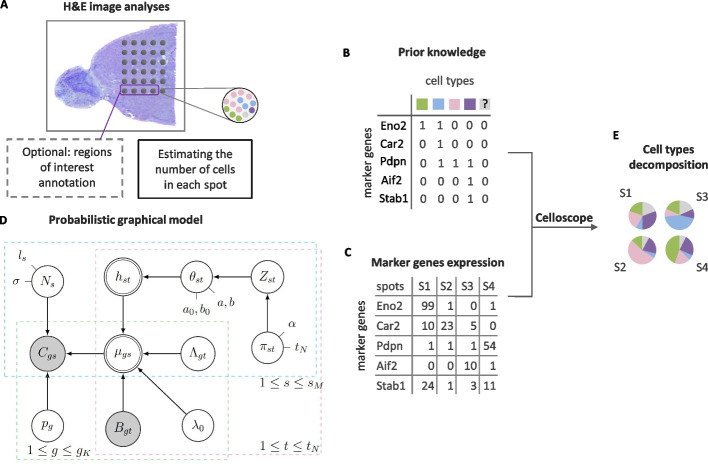
Celloscope overview. **A** The total number of cells for each spot is estimated based on a H&E image. Optionally, regions of interest are annotated. H&E image source: 10x Genomics. **B** Prior knowledge on marker genes is given as the model’s input, in a form of a binary matrix, together with ST data on marker gene expression in spots (**C**). **D** The graphical representation of Celloscope. Gray nodes correspond to the observed variables, double circled to deterministic ones, while the remaining nodes correspond to hidden variables. Arrows represent probabilistic dependencies. Model’s variables are described in Table 1 and hyperparameters in Table 2. **E** Cell type decomposition in each spot using Celloscope is performed via MCMC inference

We propose Celloscope, a novel Bayesian probabilistic graphical model of gene expression in ST data, which deconvolutes cell type composition in ST spots, and a method to infer model parameters based on an MCMC algorithm (Methods). Apart from gene expression measurements per each spot localized in the analyzed tissue, ST data come also with corresponding images of hematoxylin and eosin (H&E) staining of the same sample. The first step of the Celloscope’s pipeline is an analysis of those H&E images (Fig. 1A). In this step, the total number of cells for each spot is estimated. Optionally, regions of interest in the tissue (e.g., an inflammation) are annotated by a pathologist, so that further analysis steps are restricted to these regions.

Prior knowledge of marker genes for considered cell types is encoded as a binary matrix (Fig. 1B). The types correspond to such cells that are expected to be found in the examined tissue or (optionally) in the selected area of interest. Additionally, we assume the presence of a *dummy type* accounting for novel or unknown cell types. The *dummy type* is characterized by zero marker genes.

The estimated cell counts and the binary matrix encoding prior knowledge of marker genes, together with the measured expression of the marker genes in each (selected) spot (Fig. 1C) constitute the input to the probabilistic model (Fig. 1D). The model assumes that the measured expression depends on the hidden cell type mixture in each spot. As the output, Celloscope returns the proportions of cell types for each spot of interest (Fig. 1E).

### Celloscope’s results on simulated data prove exceptional performance of marker gene-based cell type deconvolution

To demonstrate the excellent performance of Celloscope in marker gene-based cell type deconvolution, we tested the model on different setups of simulated data, for which we knew the ground truth about the underlying cell type composition, and therefore we were able to confront the true cell type proportions across spots with the model’s estimates. We consider twelve distinct simulation scenarios which differ by two key simulation parameters. The first simulation parameter is the average number of cell types present within each spot, with the *dense* scenario implying an increased number and the *sparse* scenario denoting a decreased number of cell types per spot. The second simulation parameter controls what is given as input to Celloscope and how this information is accounted for in the model. By default, Celloscope takes cell counts in each spot and sets them as priors for the hidden variable $N_s$ (the total number of cells in spot *s*). Moreover, by default, Celloscope considers that the expression levels of marker genes in each cell type are unknown and are modeled using hidden variables $\Lambda$. In our simulations, we investigate the performance under these default settings, together with deviations from these settings. Specifically, we vary whether the number of cells in each spot is treated as *known* (i.e., given as observed variables to the model, fixing variables $N_s$), as *noisy priors*, (i.e., introduced as informative priors for the hidden variables $N_s$, with two levels of noise added: moderate or high), or as *unknown* (i.e., not given as input to the model at all―in this case uninformative priors for $N_s$ are used), and finally, whether the expression values $\Lambda$ are provided as *known* and given as observed variables to the model. For each scenario, 15 datasets were simulated, assuming 800 spots, 7 cell types (including the *dummy type*), and 149 marker genes.

**Fig. 2:**
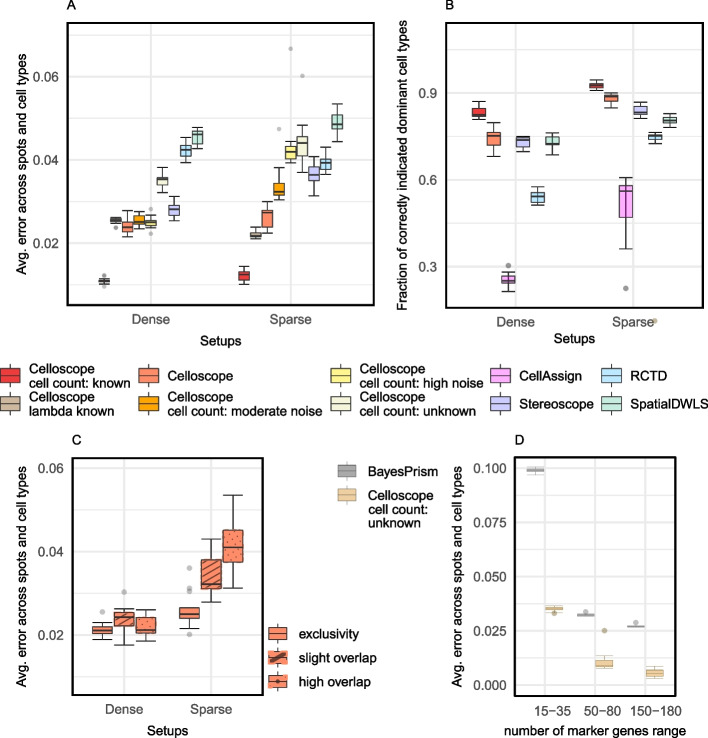
Excellent performance of Celloscope on simulated data. **A** Box plots represent distributions of the average absolute error (*y*-axis; computed using Eq. 16) for different methods (colors) and data simulation scenarios (*x*-axis). Celloscope outperforms competing methods, stereoscope [8], RCTD [7], and SpatialDWLS [13] which rely on additional input regarding gene expression levels in different cell types. Moreover, Celloscope is robust to noise in cell counting results and its performance remains satisfactory in case of no prior knowledge about the number of all cells in ST spots. **B** Distribution of the fraction of correctly identified dominant cell types across spots (*y*-axis), for different methods (colors) and simulation scenarios (*x*-axis). Celloscope again shows a large advantage over other methods. Here, we compare also to CellAssign [21], indicating the benefit from decomposing a mixture of different cell types in ST spots. **C** The impact of a lack of exclusivity in marker gene sets for cell types. Overlap in marker gene sets does not affect Celloscope’s performance in case of dense data and the performance remains satisfactory for sparse data. **D** BayesPrism [15] requires at least 50 genes per type to perform inference successfully

We first computed the average absolute error across spots for each replicate. For a single spot, the absolute error value ranges between 0 (when the model perfectly predicts the simulated fractions of the spot occupied by each cell type) and 2/*T*, where *T* denotes the number of types. The upper bound for this error can easily be seen by considering a case when the simulated fractions would be such that the spot would be fully dominated by one cell type with fraction 1, while the model would predict full domination by a different cell type. Thus, for $T=7$, we expect that the average absolute error across spots will be in the range [0, 0.29]. In this evaluation, Celloscope achieved excellent performance, with error levels between 0.01 and 0.03 (Fig. 2A) for all the aforementioned simulation settings, proving that knowledge of marker genes for cell types is sufficient for accurate cell type deconvolution in ST spots. The default Celloscope version achieved a median error of only around 0.025 for the *dense* simulation scenario and around 0.027 for the *sparse* scenario. The additional information on the number of cells in each spot increased the accuracy of the model’s prediction.

#### Celloscope is robust to noise in cell counts estimation results

Celloscope proved to be highly robust to noise in the input total number of cells in each ST spot (Fig. 2A). We considered two intensities of Gaussian noise added to the true cell counts, *moderate* *N*(2, 3) and *high* *N*(5, 5), that were randomly added or subtracted from the true value (which on average was equal to 15). In the case of the moderate noise, for both *sparse* and *dense* simulation scenarios, Celloscope manifested comparably high accuracy as in the default case of input cell counts without noise, with an average error of circa 0.025 for both scenarios. Celloscope performance remained satisfactory in the case of high noise, with an average error of 0.033 for the *dense* and 0.043 for the *sparse* scenario. Moreover, we tested Celloscope’s performance in the event of a lack of prior knowledge on cell counts; when the model is a priori completely unaware of the number of cells in each ST spot, Celloscope achieved satisfactory accuracy with an average error 0.035 for the *dense* scenario and 0.044 for the *sparse* scenario. Due to technical difficulties with H&E image quality or overlapping cell nuclei, the counts estimated for real data may be noisy, and the demonstrated robustness of Celloscope to noise in the input cell counts per spot is an important advantage.

#### Knowledge of gene expression profiles of cell types does not have a large impact on model accuracy

We next assessed the hypothetical improvement in accuracy coming from including gene expression profiles of cell types obtained from an additional, external data source, such as a scRNA-seq reference dataset. To this end, instead of estimating hidden variables $\Lambda$, as in the default setting, we run Celloscope with $\Lambda$ set to their true, simulated values (Fig. 2A). In the case of the *dense* scenario, the performance stayed at the comparable level to the default Celloscope’s setting. However, we observed some error drop for the *sparse* scenario (from median 0.027 for the default to 0.022). Still, this improvement was smaller compared to the one obtained from knowing the cell counts per spot (i.e., when the corresponding hidden variables were fixed to their true values). Note that the evaluated setup did not account for the fact that the expression profiles obtained from external data sources may have technical bias and noise. In such a case, these observations could even deteriorate the model’s performance. These results indicated that by accounting for knowledge of marker genes for each cell type and estimating the unknown expression profiles for cell types as a part of the model inference, Celloscope deals well with the lack of external data sources such as a reference RNA-seq dataset.

#### Overlapping marker gene sets do not affect Celloscope’s performance on simulated data

We further assessed whether Celloscope’s performance is sensitive to the lack of exclusivity in marker genes sets for the different cell types (Fig. 2C). To this end, we fixed the total number of marker genes to 149, as in all other simulation settings, and run Celloscope in three scenarios: (1) *exclusivity*―with no intersections between the sets of marker genes for cell types; (2) *slight overlap*―with 9 marker genes shared between cell types 2 and 3, as well as 10 shared between 3 and 4; (3) *high overlap*―in addition to the overlap from (2), with 17 genes shared between type 5 and 4, resulting in 5 out of 6 (not counting the *dummy type*, which has no markers) cell types vaguely defined. For the *dense* simulation scenarios, Celloscope is extremely robust to increasing overlap in the marker genes. For the *sparse* scenarios Celloscope’s error increases with the amount of overlap, but only slightly, reaching a median error of around 0.041 for the *high overlap* simulation setting.

#### Celloscope performs favorably over preceding approaches

We compared Celloscope’s performance to previously published Stereoscope [8], RCTD [7], and SpatialDWLS [13]. These methods, unlike Celloscope, require a reference scRNA-seq dataset to compute gene expression profiles for the analyzed cell types. Importantly, all considered methods were applied on exactly the same simulated datasets, for which the ground truth was known. Thus, we were in possession of the true marker gene expression levels across cell types, which were provided to RCTD, Stereoscope, and SpatialDWLS [13], and used by these methods for the estimation of cell type proportions in simulated spots (see Additional file 1: Sections S1, S2, and S3 for run settings used for RCTD Stereoscope and SpatialDWLS, respectively). These values were not provided to Celloscope, but inferred. Therefore, RCTD, Stereoscope, and SpatialDWLS were given a head start as compared to our model.

Despite being given such an advantage, RCTD, Stereoscope, and SpatialDWLS performed poorly compared to Celloscope (Fig. 2A). The observed much higher error for RCTD might occur due to the fact that this model uses Poisson distribution to model gene expression, in contrast to Celloscope and Stereoscope, that utilize the negative binomial distribution.

We performed a separate simulation study to compare Celloscope’s performance to BayesPrism [15], as this method requires at least 50 genes representative of each considered cell type, higher than 15–35, which we used for Celloscope and other methods in all other simulation scenarios. Importantly, for some cell types, acquiring such a high number of marker genes is unrealistic in the case of real data. In the case of setups with lower, more realistic numbers of marker genes, BayesPrism obtained a very high average error of around 0.1, much higher than Celloscope in its most difficult setup, where it was not given cell counts per spot as input (Fig. 2D). For simulated data with 50–80 and 150–180 marker genes per cell type, BayesPrism achieved better accuracy; however, it is still considerably lower than Celloscope. We also observed a lower average error achieved by Celloscope as the number of marker genes increased, despite no prior knowledge of the total number of cells per spot.

#### Accounting for cell-type mixtures in spots increases model’s accuracy

To evaluate the importance of the assumption of the presence of mixtures of cells of different types in ST spots and inferring proportions of cell types per spot, in contrast to indicating only the dominant type, we compared the results of Celloscope and other methods designed for ST data to CellAssign [21] (run settings provided in Additional file 1: Section S4). Similarly to Celloscope, CellAssign uses a binary cell type marker matrix as model input. However, CellAssign was originally developed to assign types to cells in scRNA-seq data, and as such it assumes that each observation refers to only a single cell (of a given type). Therefore, we expect that CellAssign, applied to simulated ST data, will treat each spot as homogeneous and indicate the dominant type. For each spot, we checked if the true dominant cell type (the cell type characterized by the highest proportion) was in agreement with the dominant type inferred by each method (Fig. 2B). Both Celloscope and other methods dedicated to ST and performing the cell type deconvolution performed the task of finding the dominant type significantly better than CellAssign (Fig. 2B). For CellAssign in the *dense* simulation scenario, the median fraction of correctly indicated dominant cell types was only around 0.25, while for the *dense* scenario, it was around 0.55. For Celloscope in its default setting, these measures were much higher and ranged around 0.75 and 0.88, respectively. These results confirm the key importance for accounting of the mixture of cell types in each ST spot.

### Celloscope localizes cell types in agreement with known mouse brain structures

#### Decomposition of cell types in a sagittal mouse brain section

**Fig. 3:**
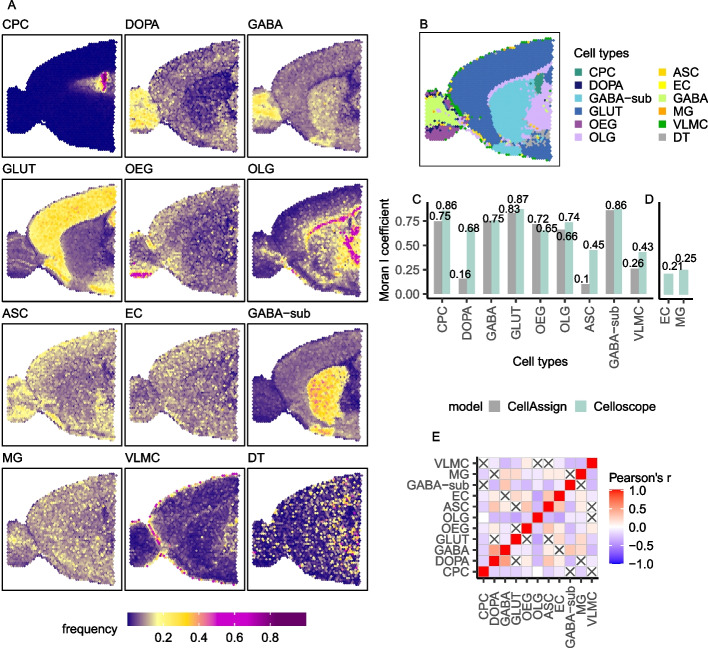
Results obtained for the anterior part of the mouse brain (sagittal section). CPC, choroid plexus epithelial cells; DOPA, dopaminergic neurons; GABA, GABAergic neurons; GLUT, glutamatergic neurons; OEG, olfactory ensheathing glia; OLG, oligodendrocytes; ASC, astrocytes; EC, endothelial cells; GABA-sub, GABAergic neurons subtype; MG, microglia; VLMC, vascular and leptomeningeal cells; DT, dummy type. **A** Heatmaps represent spatial composition for selected cell types. Dark violet indicates the absence of the cell type in question, yellow signalizes moderate occurrence, and magenta dominance of a given type. **B** Results of CellAssign on the same dataset. **C** Moran's *I* coefficient for cell types indicated both by CellAssign and Celloscope. **D** Moran's *I* coefficient computed for cell types indicated only by Celloscope. **E** The correlation matrix heatmap represents the values of the Pearson correlation coefficient for all studied cell types, the positive values in red, negative in blue. 0 indicates that there is no relationship between studied variables. “X” denotes an insignificant correlation (*p*-values of the test with the test statistics based on Pearson’s product moment correlation coefficient $p \le 0.05$)

Celloscope was applied to mouse brain data [22] and was able to successfully indicate brain structures (Fig. 3A). Specifically, an analysis of spatial transcriptomics data on sagittal mouse brain slices (an anterior section) was performed. In contrast to simulated data, for this dataset, there is no ground truth specifying the exact, underlying compositions of cell types in each spot. We can, however, expect that some of the known cell types will dominate in specific brain regions and that some other non-specific cell types will be prevalent across the entire brain tissue. Thus, to evaluate the quality of cell type deconvolution by Celloscope, we first compared the obtained spatial cell type distribution to regions as specified by the mouse brain atlas [23]. Second, we compared these findings to results of other studies localizing cell types in mouse brain regions using different technologies, namely immunofluorescence detection [24], Nissl-staining for cells and genetic marker stains [25] or labeling with anti-TH antibody [26].

Neurons and non-neuron cells called glia are the most commonly occurring brain cells [27]. Two major subclasses of neurons can be distinguished: GABAergic neurons establishing inhibitory synapses and glutamatergic neurons establishing excitatory synapses. We showed that Celloscope was able to spatially distinguish between the two of them: GABAergic neurons were found mainly in the olfactory bulb and olfactory cortex, while glutamatergic neurons were found mainly in the cerebral cortex [23]. These results were similar to those obtained in [25], albeit using a different technology.

Glia do not produce electrical impulses but rather provide support and protection for neurons. Celloscope identified that, one of the main cell types of glial cells, astrocytes, which surround and support neuron functioning, are localized throughout the entire examined sample, similarly as found in [24]. Microglia, the immune cells of the central nervous system, are constantly testing the environment for signals of malfunctioning and acting in the event of trouble. Their function justifies their omnipresence in limited quantities throughout the examined sample, as correctly identified by Celloscope.

Dopaminergic neurons synthesize the neurotransmitter dopamine. Similarly as in [26], these cells were found by Celloscope in the olfactory bulb. What is more, as expected, choroid plexus epithelial cells were localized in the choroid plexus and olfactory ensheathing glia cells were found in the olfactory bulb.

#### Comparison to CellAssign for the sagittal mouse brain section data

To show the benefits of accounting for the presence of cell type mixtures in spots as opposed to assuming they contain cells of single types, we compared the Celloscope’s outcomes to results obtained with CellAssign [21]. Since CellAssign was originally developed to assign types to single cells based on scRNA-seq data, applying this approach to ST data is equivalent to considering each spot as homogeneous with respect to cell types, as if each spot was a single cell. Similarly to Celloscope, CellAssign correctly delineates mouse brain regions, assigning spots to dominating cell types for each region (Fig. 3B). In contrast to Celloscope, however, CellAssign per construction cannot identify cell types that are present in the examined tissue in lower prevalence, such as astrocytes, endothelial cells, and microglia. For instance, while Celloscope indicates that astrocytes tend to occur in low amounts, mostly in the cerebral cortex, CellAssign omitted this cell type almost entirely, indicating only 33 spots out of 2696 to be dominated by astrocytes. Lastly, microglia endothelial cells were both identified in only six spots. On this account, we distinguished two groups of identified cell types: indicated both by Celloscope and CellAssign (Fig. 3C) and cell types that were identified only by Celloscope (Fig. 3D). In summary, the results obtained by Celloscope and CellAssign were in agreement; however, the Celloscope’s inherent feature of accounting for cell type mixtures enables it to provide a more comprehensive and more insightful description of the cell type composition of the tissue in hand.

#### Spatial autocorrelation of cell types for the sagittal mouse brain section

Given the naturally occurring tissue organization and structure, it is expected that neighboring spots will display spatial similarity and cells of the same type will co-localize. Note that Celloscope treats all spots as independent, regardless their position and potential proximity. As a consequence, spatial correlation across spots is not enforced in the model and can be used to validate the model’s performance. To this end, we calculate the Moran's *I* coefficient [28, 29] to quantify the level of spatial autocorrelation of inferred cell type proportions (Fig. 3C, D). The Moran’s *I* coefficient takes values from $-1$ to 1, where $-1$ indicates perfect dispersion, 0 perfect randomness (no autocorrelation), and 1 perfect clustering of homogeneous values. Therefore, high values of the Moran's *I* coefficient indicate that the inferred cell types cluster in space. We observe very high spatial autocorrelation for the majority of cell types and moderate for microglia and endothelial cells; however, in all cases, the obtained spatial autocorrelation is non-negligible. Note that the level of spatial autocorrelation for Celloscope is similar to the autocorrelation level obtained by CellAssign, despite the fact of solving a more demanding and cumbersome task of cell type deconvolution as opposed to assigning the dominant cell type to a spot. Importantly, those cell types that were found in the tissue only by Celloscope and not by CellAssign also show spatial autocorrelation.

#### Spatial co-occurrence and mutual exclusivity between cell types for the sagittal mouse brain section

The cell type composition of spots resolved by Celloscope allows investigating spatial co-occurrence and exclusivity of cell types (Fig. 3E). We find that GABAergic neurons tend to spatially co-occur with dopaminergic neurons and GABAergic neurons subtype and glutamatergic neurons with astrocytes. On the other hand, Celloscope results suggest that oligodendrocytes avoid co-localizing with dopaminergic, glutamatergic and GABAergic neurons.

#### Comparison to STdeconvolve for the sagittal mouse brain section

Furthermore, we compared Celloscope’s performance to STdeconvolve [16], which also does not require a reference in a form of scRNA-seq data to estimate cell type expression profiles. In contrast to Celloscope, the inference in STdeconvolve is not guided by any prior knowledge. Instead, this model works in a fully unsupervised manner and discovers latent *topics* in gene expression, which are interpreted as cell types. These identified topics are not annotated and need to be further interpreted to assign some specific cell type to each topic. To this end, the transcriptional profiles of the topics inferred by STdeconvolve should be compared to known transcriptional profiles of specific cell types, or gene set enrichment analyses should be performed based on a list of reference gene sets for different cell types.

When applied to the sagittal mouse brain section data, STdeconvolve (see Additional file 1: Section S6 for run setting) identified 10 topics, which in the majority of cases by visual inspection of their spatial localization could be matched to a subset of 12 cell types found also by Celloscope (Additional file 1: Fig. S1). However, while all but one topic identified by STdeconvolve form evident, specific spatial structures, Celloscope indicated three cell types, which did not manifest such a behavior (astrocytes, endothelial cells and microglia). The omnipresence of these three cell types across the whole brain tissue is justified by their function and was reported previously [25]. For example, endothelial cells form the lining of your blood vessels that entwine the whole brain [30]. This suggests that topics found by STdeconvolve can potentially correspond not to a single but several types of cells that happen to be present in the same region of the tissue.

Moreover, Celloscope distinguished between vascular and leptomeningeal cells and olfactory ensheathing glia, in contrast to STdeconvolve that seemingly merged those two cell types (annotation of the corresponding topics was performed here by matching the spatial localization of known cell types found by Celloscope to the unannotated topics found by STdeconvolve).

#### Celloscope’s results obtained for mouse brain coronal section data are in agreement with the results for the sagittal section

**Fig. 4:**
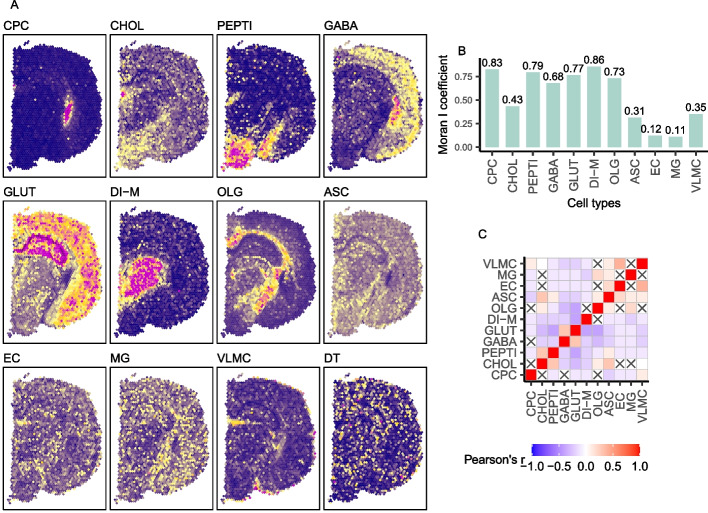
Results obtained for the anterior part of the mouse brain (coronal section). OLG, oligodendrocytes; OEG, olfactory ensheathing glia; ASC, astrocytes; GABA, GABAergic neurons; GLUT, glutamatergic neurons; DI-M, di-mesencephalon neurons; CPC, choroid plexus epithelial cells; EC, endothelial cells; MG, microglia; VLMC, vascular and leptomeningeal cells; CHOL, cholinergic neurons; PEPTI, peptidergic cells; DT, dummy type. **A** Heatmaps represent spatial composition for selected cell types. Dark violet indicates the absence of the cell type in question, yellow signalizes moderate occurrence, and magenta dominance of a given type. **B** Moran's *I* coefficient for cell types. **C** The correlation matrix heatmap represents the values of the Pearson correlation coefficient for all studied cell types, the positive values in red, negative in blue. 0 indicates that there is no relationship between studied variables. “X” denotes an insignificant correlation (*p*-values of the test with the test statistics based on Pearson’s product moment correlation coefficient $p \le 0.05$)

Further, we performed an analysis of spatial transcriptomics data for a coronal mouse brain slice [31] that is orthogonal to the sagittal section analyzed above (Fig. 4A). As we were analyzing the same tissue type, we were locating the same cell types, namely oligodendrocytes, astrocytes, GABAergic neurons, glutamatergic neurons, choroid plexus epithelial cells, endothelial cells, microglia, and vascular and leptomeningeal cells. However, since in our previous analyses (Fig. 3A) a portion of cells was assigned to the *dummy type*, we also considered two additional cell types, i.e., cholinergic neurons, peptidergic cells, and di-mesencephalon neurons. The markers for the considered cell types were found using the marker identification procedure based on lead genes (Methods), taking candidate marker genes from the dataset of Zeisel et al. [32].

We observed consistency in cell type composition inferred by Celloscope between the two sections, particularly for choroid plexus epithelial cells, oligodendrocytes, and glutamatergic neurons. Similarly, as for the sagittal section, we found that microglia, astrocytes, and endothelial were omnipresent throughout the entire investigated sample. Finally, we found that the spatial localization of di-mesencephalon neurons and peptidergic cells inferred by Celloscope agreed with their known position in the mouse brain.

Again, we computed the Moran's *I* coefficient to quantify the level of spatial autocorrelation of the inferred cell types’ proportions (Fig. 4B). Very high values of the Moran's *I* coefficient were acquired for the vast majority of cell types (choroid plexus epithelial cells, GABAergic neurons, glutamatergic neurons, oligodendrocytes, and peptidergic cells), indicating that the inferred cell types clustered in space. Further, we investigated spatial co-occurrence and exclusivity of cell types (Fig. 4C), finding that cholinergic neurons tend to co-occur with peptidergic cells, while glutamatergic neurons tend to avoid peptidergic cells and oligodendrocytes.

As an additional validation, we considered two subtypes of the glutamatergic neuron cell type, assuming candidate marker genes reported in an independent study [32], different from the study of Ximerakis et al. [33] that we used to find the markers for the glutamatergic neuron cell type. To this end, we constructed a new prior cell type-marker matrix, now including the three *subtypes* instead of the glutamatergic neuron cell type. As expected, the different subtypes occupy distinct sub-regions of the mouse brain (Additional file 1: Fig. S2), and their total abundance agrees with the localization of the glutamatergic neuron cell type. This analysis indicates that our method is returning consistent results irrespectively of the collection of marker genes and the original source of the candidate genes for markers.

#### Comparison to STdeconcolve and SpatialDWLS for the coronal mouse brain section

We applied STdeconvolve [16] to the coronal mouse brain section data (for run setting see Additional file 1: Section S6). The obtained topics visibly cluster in space; however, again as in the case of sagittal section data, the results lack topics that would correspond to omnipresent cell types that are expected due to their function in the mouse brain, such as microglia and endothelial cells (Additional file 1: Fig. S3). This dataset was also previously analyzed with SpatialDWLS [13]. In contrast to Celloscope, SpatialDWLS benefits from using a reference scRNA-seq dataset. Despite this handicap, this method reported similar subtypes as found by Celloscope, with small differences. For example, SpatialDWLS reported slightly different spatial localization of granule neurons (Additional file 1: Fig. S2).

#### Sensitivity to the choice of marker genes

Finally, we also investigated Celloscope’s sensitivity to the choice of marker genes by running it with the same run settings on the sagittal mouse brain section but different sets of marker genes (Additional file 1: Figs. S4 and S5). These four different marker genes sets were acquired with four different sets of thresholds for the marker gene selection procedure (Methods). These thresholds are used to ensure correlation of the found marker genes with given lead marker genes for each cell type. As the values of the thresholds decrease and the procedure becomes less restrictive, the number of selected genes rises. Consequently, the found marker genes increasingly overlap between the cell types. Still, the acquired results for the different thresholds were generally in agreement, with only minor differences, proving Celloscope is insensitive to changes in marker gene sets for high quality data.

### Celloscope elucidated the source of inflammation in a human prostate tissue

**Fig. 5:**
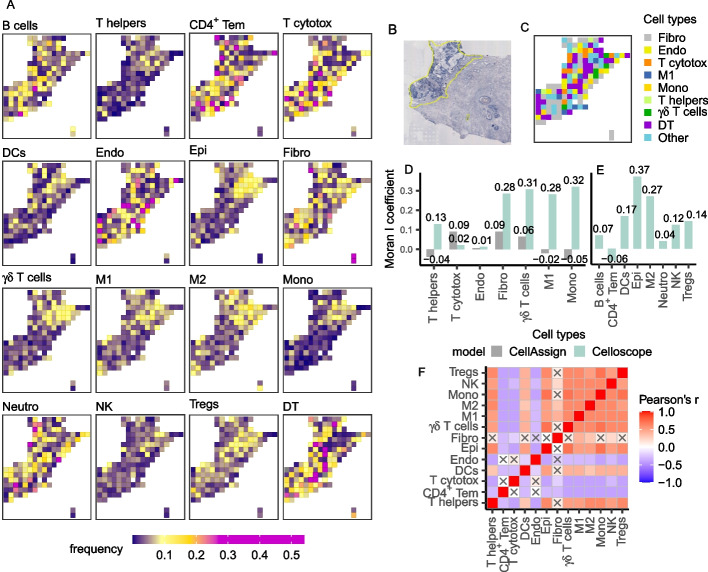
Results obtained for data from the human prostate (section 3.1). T helpers―CD4^+^ T cells (helper T cells), CD4^+^ Tem―CD4^+^ effector memory T cells, Cytotox―cytotoxic CD8^+^ T cells, DCs―dendritic cells, M1―M1 macrophages, M2―M2 macrophages, Mono―monocytes, Neutro―neutrophils, NK―natural killer cells, Tregs―natural CD4^+^ regulatory T cells, Endo―endothelial cells, Epi―epithelial cells, Fibro―fibroblasts, DT―dummy type. **A** Heatmaps represent spatial composition of cell types across spots. Dark violet indicates the absence of the cell type in question, yellow signalizes moderate occurrence, and magenta dominance of a given type. **B** The inflamed region of interest annotated on the H&E image (yellow selection). **C** Results of CellAssign performance. Colors correspond to cell types. Other―cell types that were indicated by CellAssign in not more than 4 spots, namely B cells, dendritic cells, epithelial, M2 macrophages, CD4^+^ effector memory T cells, natural CD4^+^ regulatory T cells, NK cells, and neutrophils. **D** Moran's *I* coefficient computed for cell types indicated both by CellAssign and Celloscope. **E** Moran's *I* coefficient computed for types indicated only by Celloscope. **F** The correlation matrix heatmap represents the values of the Pearson correlation coefficient for all studied cell types, the positive values in red, negative in blue. 0 indicates that there is no relationship between studied variables. “X” denotes an insignificant correlation (*p*-values of the test with the test statistics based on the Pearson’s product moment correlation coefficient $p \le 0.05$). B cells and neutrophils were disregarded as those two cell types indicated only insignificant results

**Fig. 6:**
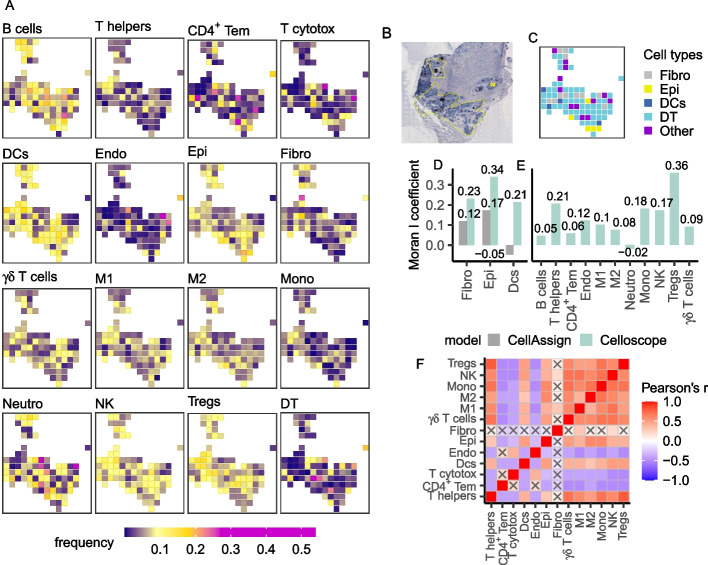
Results obtained for data from the human prostate (section 4.2). T helpers―CD4^+^ T cells (helper T cells), CD4^+^ Tem―CD4^+^ effector memory T cells, Cytotox―cytotoxic CD8^+^ T cells, DCs―dendritic cells, M1―M1 macrophages, M2―M2 macrophages, Mono―monocytes, Neutro―neutrophils, NK―natural killer cells, Tregs―natural CD4^+^ regulatory T cells, Endo―endothelial cells, Epi―epithelial cells, Fibro―fibroblasts, DT―dummy type. **A** Heatmaps represent spatial composition of cell types across spots. Dark violet indicates the absence of the cell type in question, yellow signalizes moderate occurrence, and magenta dominance of a given type. **B** The inflamed region of interest annotated on the H&E image (yellow selection). **C** Results of CellAssign performance. Colors correspond to cell types. Other―cell types that were indicated by CellAssign in not more than 4 spots, namely helper T cells, CD4^+^ effector memory T cells, cytotoxic CD8^+^ T cells, M1 macrophages, M2 macrophages, monocytes, neutrophils, natural killer cells, regulatory T cells, and endothelial cells. **D** Moran's *I* coefficient computed for cell types indicated both by CellAssign and Celloscope. **E** Moran's *I* coefficient computed for types indicated only by Celloscope. **F** The correlation matrix heatmap represents the values of the Pearson correlation coefficient for all studied cell types, the positive values in red, negative in blue. 0 indicates that there is no relationship between studied variables. “X” denotes an insignificant correlation (*p*-values of the test with the test statistics based on the Pearson’s product moment correlation coefficient $p \le 0.05$). B cells and neutrophils were disregarded as those two cell types indicated only insignificant results

Next, we applied Celloscope to analyze human prostate data [20]. The analyzed dataset contained twelve sections from different regions of a resected prostate, which were profiled using ST. Several of these sections contained cancerous tissue. We selected two sections (3.1: Fig. 5B; 4.2: Fig. 6B), where infiltrations of immune cells were visible in their respective H&E images. We applied Celloscope to investigate whether those infiltrations could be associated with an ongoing tumorigenesis in these areas, as was observed by [34, 35], or whether it was due to some other inflammatory process. Notably, this information could not be derived from the H&E image alone, as the fine subtypes of the detectable cells were not distinguishable visually. For example, mononuclear cells with abundant, *foamy* cytoplasm indicating macrophages or cells with multilobed nucleus indicating neutrophils could be detected in H&E, but it was not possible to distinguish subtypes of lymphocytes (eg., T cells, B cells or NK cells). Similarly, raw gene expression data, measured using ST in this area, was not directly indicative of the type of the visible inflammation. Since detailed dissection of infiltrating immune cell identity is not feasible with classical histopathological inspection, nor directly from ST measurements, computational tools, such as Celloscope, are required to fill this gap.

To resolve the immune cell composition in those regions, we aimed at identifying the following immune cell types across spots: B cells, CD4^+^ T cells (helper T cells), CD4^+^ effector memory T cells, cytotoxic CD8^+^ T cells, dendritic cells, $\upgamma \updelta$ T cells, M1 and M2 macrophages, neutrophils, monocytes, natural killer cells, and natural CD4^+^ regulatory T cells (Tregs). However, we also took into consideration non-immune cells that are expected to be present in the prostate tissue: endothelial cells, epithelial cells, and fibroblasts.

Celloscope identified that the immune cell type composition of spots in both sections (Figs. 5A, 6A) is characterized by a larger heterogeneity as compared to the mouse brain tissue. In general, indicating the dominant type within each spot is more difficult, since for every spot (with only a few exceptions) the dominant type takes much less than half of a spot capacity, the average dominant type has the proportion of approximately 24%.

Although the analyzed two sections are separated within the prostate, the results obtained with Celloscope indicate similarities in their cellular composition. Celloscope identified the expected mutual arrangement of cell types such as general co-existence of epithelial, endothelial, and stromal cells (fibroblasts) which compose the prostate gland. Endothelial cells, as expected, were found in the regions of exudate, as well as in areas dominated by blood vessels. Fibroblasts are enriched in the regions without visible exudate. Infiltrating immune cells, as expected, were found to be enriched in the exudate regions. The found various immune cell populations (e.g. CD4^+^ helper T cells, $\upgamma \updelta$ T cells, CD8^+^ cytotoxic T cells, macrophages, NK cells and neutrophils) were in agreement with previously observed highly heterogeneous composition of the immune infiltrate in the inflamed prostate [36, 37]. Among immune cells identified by Celloscope, neutrophils, NK cells, and dendritic cells predominated and their dispersed distribution was in agreement with the morphological features characteristic for acute inflammation.

The composition of cell types identified using Celloscope allowed us to conclude that in those two particular regions of the resected prostate, the inflammation was not caused by tumorigenesis but rather by an infection. As both examined regions were in the periurethral part of the prostate, the acute inflammation was most likely caused by a bacterial infection originating from the urethra. Identification by Celloscope of both neutrophils and NK cells in this area strongly suggests mixed infection with extra- and intracellular bacteria. There were, however, some differences in the cellular composition between the two probed sections: in the second one there is an enrichment in the regulatory T cells, which is indicative of persistent inflammation.

#### Comparison to CellAssign

We compared Celloscope’s outcomes to results obtained using CellAssign. In the case of human prostate section 3.1 (Fig. 5C) CellAssign ignored numerous cell types (B cells, CD4^+^ effector memory T cells, dendritic cells, epithelial cells, M2 macrophages, neutrophils, NK cells, and natural CD4^+^ regulatory T cells) and assigned each to only four or fewer spots. For those cell types that were identified both by Celloscope and CellAssign, their inferred localizations differ between the two approaches. For example, Celloscope identified that both helper T cells and monocytes are clustered in the upper part. In contrast, CellAssign found these cell types as scattered across the entire region. In the case of section 4.2 (Fig. 6C), CellAssign failed to indicate the dominant type, ignored even more cell types as compared to section 3.1 and assigned as many as 62% spots as the *dummy type*.

#### Spatial autocorrelation of cell types

To systematically quantify the extent to which cell types found using Celloscope and CellAssign cluster in space, we applied the Moran's *I* coefficient, assessing spatial autocorrelation in the results for both human prostate sections (Figs. 5D, E and 6D, E). Since we expected spatial similarities across spots in proximity and neither Celloscope nor CellAssign assumes that this is the case and treats the spots as independent, spatial autocorrelation provides the means for an independent validation for models’ results. We observed moderate spatial autocorrelation for the majority of cell types, which was consistently higher for Celloscope than for CellAssign for cell types identified by both methods with just one exception (Figs. 5D and 6D). Moreover, for the majority of cell types that were ignored by CellAssign, such as macrophages M2 (Fig. 5E) or regulatory T cells (Fig. 6E), Celloscope again indicated a clustering of spots containing those cell types.

#### Spatial co-occurrence and mutual exclusivity between cell types

Finally, we inspected patterns of spatial co-occurrence and mutual exclusivity of cell types found using Celloscope (Figs. 5F and 6F). We found that CD4^+^ helper T cells, $\upgamma \updelta$ T cells, regulatory T cells, and monocytes tend to co-localize. The largest positive spatial correlation was observed between regulatory T cells and CD4^+^ Tem. Since monocytes can differentiate into macrophages, it is not surprising that we observed a high correlation between monocytes and macrophages M1, as well as M2.

#### Comparison to STdeconvolve for human prostate data

Further, we applied STdeconvolve (Additional file 1: Fig. S6) to human prostate data, section 3.1, to compare the resulting cell type decomposition to the results of Celloscope. STdeconvolve identified only two cell types that co-localized in the upper part, while Celloscope decomposed this region further. Again, as in the case of mouse brain data, consecutive analyses would be required to assign cell types in the output of STdeconvolve. This is in contrast to Celloscope, which either automatically identifies the found cell types based on the marker gene matrix or assigns an uncharacterized, *dummy* cell type.

#### Comparison to methods designed for immune cell type decomposition in bulk RNA-seq

Finally, we compared the results obtained by Celloscope for the human prostate data, section 3.1, to the results obtained by methods designed for immune cell type decomposition in bulk RNA-seq data (using the function deconvolute from the R package immunedeconv [38]). The four compared methods included ABIS [39], EPIC [40], quanTIseq [41], and xCell [42]. We did not include MCP counter [43] in this analysis, as this method provides scores in arbitrary units that are only comparable between samples, but not between cell types. Thus, the output of this method does not allow to perform decomposition into proportions occupied by the different cell types.

Firstly, we evaluated the ability of the four methods to decompose each individual spot of ST data into cell types. To this end, we considered each ST spot as a single bulk RNA-seq sample and applied each method separately to each spot. In this analysis, ABIS failed to reliably decompose the spots. Specifically, the returned matrix of cell type fractions found across spots contained 58% negative entries. In the case of EPIC, an average of 66% of the spots were assigned to an uncharacterized cell type. These issues encountered by ABIS and EPIC could be due to high technical or biological variability of data across spots [44]. Results obtained using quanTIseq and xCell were in disagreement, e.g., quanTIseq indicated B cells and Tregs in larger fractions, while xCell almost completely disregarded those two cell types (Additional file 1: Fig. S7). The results of quanTIseq are in better agreement with Celloscope than the results of xCell, although both these approaches failed to spatially group some of the cell types that were found to be spatially correlated by Celloscope. The differences between quanTIseq, xCell, and Celloscope were also due to the fact that both quanTIseq and xCell operate on their own, fixed signature matrices of marker genes for each cell type, and do not take custom matrices as input. Thus, the marker genes for xCell and quanTIseq were not guaranteed to be even expressed in non-negligible amounts across the analyzed ST spots.

Secondly, we applied these four approaches to cell type decomposition to *pseudo bulk* data for the same human prostate section. To this end, gene expression was summed across spots for every gene. In this case, ABIS again returned a large number of negative values for the cell type proportions, indicating that this approach was unable to cope with technical or biological variability also for pooled data. xCell failed to run on the pooled data, returning errors. EPIC and quanTIseq returned similar proportions of cell types, with only small differences (Additional file 1: Fig. S8). This result confirms the usability of these two approaches to bulk RNA-seq data, which has much higher coverage than the coverages for the individual ST spots.

Taken together, the comparison to the bulk RNA-seq data deconvolution approaches revealed that in contrast to Celloscope, these approaches may be applied to bulk data but not to deconvolve individual ST spots.

### Comparison of model performance and data quality between the mouse brain and the prostate cancer datasets

The convergence diagnostics using the Gelman-Rubin method showed high evidence for the convergence of Celloscope’s sampling procedure for both mouse brain and prostate cancer data (Additional file 1: Fig. S9). Still, in-depth analysis of the data quality and the inferred model parameters indicated a much higher quality of the mouse brain dataset in comparison to the prostate dataset.

Specifically, the Kendall and Pearson correlation coefficients between the expression of the lead genes (Methods) and the rest of the marker genes were much higher for the mouse brain data (Additional file 1: Fig. S10 A), as compared to the prostate data (Additional file 1: Fig. S10 B). Therefore, for the prostate data, the agreement of gene expression among the marker genes for the cell types was lower and the model had a harder task identifying where the cell types were located. Second, a comparison between average gene expression per spot between mouse brain and prostate cancer data showed much lower coverage for the latter (Additional file 1: Fig. S10 C); therefore, the model receives a much weaker signal in case of the human prostate data. Finally, the inferred $p_g$ parameters for genes in the prostate data, corresponding to the variance of the gene expression values, obtained much higher values for prostate than for the mouse brain data (Additional file 1: Fig. S10 D), indicating much higher over-dispersion in the prostate data. Those issues suggest that the results obtained for the human prostate should be approached with more caution than those obtained for the mouse brain data.

### Run-times and computational complexity of Celloscope

On a system equipped with AMD Ryzen Threadripper 3990X 64-Core CPU and 128 GB RAM, using 16 CPUs, 10,000 iterations for human prostate data (274 spots, 173 genes, 16 cell types) took 407.29 s. On the same system, 10,000 iterations for mouse brain data (2696 spots, 179 genes, 12 cell types) took 1 h 45 min.

Computational complexity (the number of performed operations) depends on the number of iterations until convergence and the number of parameters, that is dependent on the number of ST spots, the number of marker genes, and the number of cell types considered. In each iteration of the sampler, we either draw a sample from the posterior or perform an accept-reject step. Both of them require computing values of probabilities densities/mass functions in certain points (vectorized operations in SciPy [45]). Additionally, accept-reject steps require drawing random numbers from predefined probability distributions (inbuilt functions in SciPy or inverse transform samplers). Calculating parameters of those distributions requires matrix multiplication (in NumPy [46]), which is the major operation with the highest computational cost, taking into consideration all steps of the algorithm. Dimensions of the multiplied matrices are (*K*, *N*) and (*N*, *M*), where *K* denotes number of marker genes, *M* denotes number of spots and *N* denotes number of cell types. Thus, the pessimistic computational complexity of the algorithm can be estimated as $\mathcal {O}(I\cdot K\cdot N\cdot M)$, where *I* is the number of the iterations of the sampler. The number of independent chains does not affect computational complexity, as independent runs are paralleled with the use of Python functions Parallel and delayed from the Python package joblib.

## Discussion

The results of our extensive simulation study demonstrated the Celloscope’s accurate performance. Moreover, the outcomes of the conducted analyses of mouse brain data showed the Celloscope’s capability to unravel spots’ percentage composition with respect to cell types. Finally, the obtained insight into the source of inflammation in the human prostate dataset showed the Celloscope’s usefulness to investigate heterogeneous tissue’s functionality. The correctness and effectiveness of our approach are additionally demonstrated by the high spatial correlation for inferred cell type proportions measured with the use of the Moran's *I* coefficient.

Celloscope not only has numerous, significant advantages, and shear strengths; it also substantially stands out from previously proposed methods that rely on integrating scRNA-seq with ST data. The fact that Celloscope is fully independent of scRNA-seq data intrinsically mitigates risks encountered while integrating data from the two disparate platforms. Further, we explicitly account for the total number of cells (regardless of their type) present at each ST spot by introducing a dedicated random variable to the model. This variable is particularly important as the number of cells may vary significantly across spots and extensively influence gene expression level measured for each spot. Therefore, accounting for the number of cells in each spot significantly boosts inference accuracy. Moreover, the Bayesian approach allows to incorporate additional information, such as the probability of a certain type being present at a certain spot, as priors.

Our results strongly advocate for the need to account for cell type mixtures in ST spots instead of assigning whole spots to single types. The advantage of accounting for mixtures was made evident by confronting our results with results obtained with CellAssign, a tool designed to assign types to single cells. On simulated data, CellAssign achieved much lower performance in terms of identifying the dominant type in spots. In application to prostate inflammation data, the Celloscope’s results are characterized by a significantly higher Moran's *I* spatial correlation coefficient as compared to CellAssign.

There are, however, certain limitations as far as applying Celloscope is concerned. The use of prior knowledge on marker genes naturally restricts the scope of considered cell types to a predetermined, closed list. To address this issue, we introduce the *dummy type* to account for the presence of other cell types than listed. As the Celloscope’s inference is based solely on marker gene sets, our method requires human attention and expert knowledge, which may be demanding to acquire. Additionally, Celloscope’s ability to distinguish between cell types is limited to these cell types for which marker genes are determined, and, as a result, the rest of cell types may not be identified by Celloscope.

Finally, our approach does not account for and could be extended to explicitly incorporate spatial coordinates of spots during modeling and inference. In this way, the neighboring spots could borrow statistical strength from one another as, generally, one expects similarities in cell-type composition between nearby spots. This could be achieved, for example, using a hidden Markov random field (HMRF) as in STARCH [47] (an approach for inferring copy number alterations in ST data) and as in FICT [48] (an approach for assigning cell types in spatial single-cell expression data) and constitutes future work for this project.

As the number of analyzed high-throughput sequencing datasets will grow, our current understanding of cell types and marker genes will expand even more. This will trigger the growth and strengthen the importance of marker-gene-driven methods such as Celloscope. In the current study, Celloscope was applied to localize different cell types, as defined by their marker genes. It is worth noting that the same framework is readily applicable to localizing cell types in different states. Indeed, we can also use marker genes to define cell states, which can be thought of as laying one step lower in the hierarchical structure of cell types. For example, Celloscope could be applied not only to identify spots harboring B cells, but also to separate them into *activated cells B cells* and *resting B cells*.

In a broader context, let us notice that Celloscope can be perceived as a probabilistic framework for describing and modeling the signal conveyed by a mixture of different factors or entities (in our case, cell types) and could be used in other deconvolution problems.

## Conclusions

In this contribution, we proposed a probabilistic Bayesian framework for comprehensive and accurate decomposition of cell type mixtures in ST spots based on marker genes. In this way, we enable spatial mapping of known cell types and indication of the presence of novel ones in tissues examined using the ST technology. Importantly, we are able to complete this task without a reference scRNA-seq dataset. However, we do incorporate prior domain knowledge about cell types and their corresponding marker genes, with the aim to limit the hypothesis space and guide toward feasible solutions [17, 18]. Thus, our method benefits from knowledge that has been accumulated throughout many years, via different techniques and by independent researchers [19]. In summary, Celloscope is a step forward in developing new probabilistic methods used to analyze spatial transcriptomics data and answer diverse biological questions.

## Methods

### Analyzed data

#### Mouse brain datasets

ST profiling of the anterior part of the mouse brain tissue sagittal section and mouse brain coronal sections was generated with the Visium technology from 10x Genomics. Raw data can be downloaded from [22, 31]. The analyzed count matrices [22] are outputs of the spaceranger pipeline [49]. Sagittal data was accessed via SeuratData package [50, 51] (Additional file 1: Section S7). The sagittal dataset contains expression of 31,053 genes in 2,696 spots, given by read counts. One hundred seventy-nine genes were selected and modeled as markers (see below). Across the spots, a minimum of 14, on average 119, and a maximum of 153 genes had non-zero expression. The minimal total expression per spot was 18, maximal was 13,788, while average total expression was 1199.061. Coronal data was downloaded from [13] and contains expression of 31,053 genes in 2,698 spots. Four hundred thirty genes were selected and modeled as markers (see below). Across the spots, a minimum of 27, on average 252, and a maximum of 354 genes had non-zero expression. The minimal total expression per spot was 35, maximal was 27,993, while average total expression amounted to 2,239.

#### Human prostate dataset

The analyzed human prostate data was generated by Berglund et al. [20]. The raw files generated with ST were preprocessed by the authors as described in [52]. The data comprised twelve sections from different regions of a resected prostate affected by cancer. We restricted our analyses to spots that were contained in an area annotated as inflamed by an expert pathologist based on the respective H&E images. We present results for 140 spots from section 3.1 and 87 from section 4.2. A semi-automatic procedure selected 173 genes as marker genes (see below). Across the analyzed spots, a minimum of 7, on average 88, and a maximum of 163 genes had non-zero expression. The minimal total expression per spot amounted to 21, maximal was 5150, while the average total expression was 921.7.

### Defining the marker genes for cell types

Celloscope expects as input a binary matrix specifying marker genes for each cell type. This matrix reflects prior knowledge and should be curated manually by the user. To make the task of determining the specific marker genes for each cell type easier for the user, Celloscope implements a semi-automatic procedure of marker selection. Importantly, the procedure does not use scRNA-seq data, therefore Celloscope remains independent of a reference in the form of a scRNA-seq dataset.

More specifically, the input to the marker selection procedure is given by the following: (i) sets of (possibly large) candidate marker genes for each cell type―these sets could be collected from published datasets and literature, (ii) threshold $r_N$ for the number of spots a marker gene should be expressed in (five by default), (iii) threshold $r_K$ for marker gene expression level, (iv) for each cell type *t*, a single *lead gene* $g^*_t$- a core marker gene with high expression in the analyzed ST data which the user is sure that it definitely is representative of *t*, i) threshold for normalized Kendall correlation $\tau ^*_t$ and for normalized Pearson correlation $\rho ^*_t$. The selection procedure acts as a ”sieve” for the candidate genes and retains marker genes with good quality expression signal in the analyzed ST data, and for each cell type, it aims at finding such marker genes that are co-expressed with the *lead gene* for that type within the same ST spots. In this way, the effort of the user is reduced to careful specification of a single *lead gene* per each cell type, and collecting large input sets of candidate genes, without caring about the quality of each individual candidate, leaving their selection to the procedure.

To this end, the procedure involves the following steps:

1. For each cell type *t*, retain candidate genes that were expressed in more than $r_N$ spots at expression level higher than $\text {r}_K$, and store the retained candidates in a gene set $R_t$. This step filters out candidate marker genes that are characterized by low expression in the analyzed data.
2. For each cell type *t* and for each $g \in R_t$, compute $\tau$-Kendall correlation $\tau _g^t = \tau (C_{g^*_t : }, C_{g : }$) and Pearson correlation $\rho _g^t = (C_{g^*_t : }, C_{g: })$ of that gene *g* with the lead gene $g^*_t$, where $C_{gs}$ denotes gene expression of gene *g* in spot *s* and $C_{g:}$ denotes the vector of expression of *g* across all spots.
3. Normalize vectors $\tau _g^t$ for ${g \in R_t}$ and $\rho _g^t$ for ${g \in R_t}$ by their maximal values.
4. For each cell type *t*, select the final marker genes that satisfy the condition $\tau _g^t > \tau ^*$ AND $\rho _g^t> \rho ^*$. This step retains marker genes coexpressed with the lead gene across ST spots.

Candidate marker genes for mouse brain data (the sagittal section) were taken from [33]. Genes that served as lead genes were as follows: *Th*, *Fabp7*, *Cck*, *Meis2*, *Ppp1r1b*, *Plp1*, *Ttr*, *Gja1*, *Cldn5*, *Hexb*, *Ptgds*. The output of the marker gene selection procedure is available in Additional file 2. Candidate marker genes for mouse brain data (the coronal section) were taken from [32, 33]. Genes that served as lead genes were as follows: *Cck*, *Ppp1r1b*, *Plp1*, *Ttr*, *Gja1*, *Cldn5*, *Hexb*, *Ptgds*, *Sncg*, *Dlk1*, *Prkcd*, *Pde1a*, *Nr3c2*. The output of the marker gene selection procedure is available in Additional file 3. Candidate marker genes for human prostate data were curated from [42, 53–59] and based on expert knowledge. Genes that served as lead genes were as follows *DUSP4*, *CD3E*, *SRC*, *UBE2B*, *BRD2*, *DDX5*, *GGA2*, *PFN1*, *TRAC*, *VWF*, *DCN*, *CDH1*, *HLA-DRA*, *HLA-DRB1*, *CXCL8*. Genes assigned as markers to more than 2 cell types were removed. Again, the output of the marker gene selection procedure is available in Additional file 4.

### Annotation of areas of interest based on the H&E image for the human prostate sample

In order to assess the type of tissue present in each spot for the human prostate dataset, we annotated contiguous tissue regions using QuPath [60]. The annotated tissue types were as follows: suspected cancer, cancer, immune cells: chronic inflammation, immune cells: acute inflammation and suspected acute inflammation. To obtain lists of spots per each annotated area, we overlapped spot coordinates with tissue annotations using a custom script in QuPath.

### Cell counting

We estimated the number of cells in each spot using a dedicated, custom script extending upon the functionality of QuPath [60]. First, areas occupied by the circular spots in the analyzed H&E images were identified by their coordinates and diameter. Then, cell nuclei placed within these circular spot areas were detected using QuPath’s inbuilt cell counting algorithm for H&E image analysis (function WatershedCellDetection). To enssure the counted cells were correctly distinguished from the background noise in the images by the algorithm, we manually, carefully adjusted the algorithm’s parameters, so that the returned cell counts in randomly selected spots were in agreement with counting cells by eye. The cell counting procedure was performed for all spots on the mouse brain slide and for the pathologist-selected inflammation area for the human prostate dataset.

### The Celloscope model for cell type deconvolution in ST data

Celloscope is a novel, probabilistic, hierarchical Bayesian model of gene expression in ST data that can be used for marker gene-driven estimation of the proportions of different cell types present at selected spots of the examined tissue (Fig. 1). Celloscope represents all variables of interest as random variables (see Fig. 1B for graphical representation of the model, Table 1 for the list of variables and Table 2 for the hyperparameters). Let $s \in \{s_1, s_2, \dots , s_{M} \}$ index spots. We assume $t_N$ cell types are present in the considered tissue, indexed by $t \in \{t_1, t_2, \dots , t_{N}\}$. Each type is represented by the set of marker genes. Those sets are potentially overlapping, but ideally, to better differentiate between the cell types, they should be disjoint. Let $g \in \{g_1, g_2, \dots g_{K} \}$ be a set of marker genes. A binary marker gene signature for each cell type is encoded in a matrix *B*, such that an entry $B_{gt}$ takes the value 1 if a gene *g* is a marker for a type *t* and 0 otherwise. The matrix *B* is considered prior knowledge and is modeled as an observed variable.

**Table 1:** Observed, hidden and deterministic variables in Celloscope, their corresponding probability distributions and interpretations

Observed variables		
$C_{gs}$	Negative binomial	Gene expression for gene *g* in spot *s*
$B_{gt}$	-	Indicator whether gene *g* is a marker for cell type *t*
Hidden variables		
$\theta _{st}$	Gamma	Unnormalized abundance of cell type *t* in spot *s*
$\lambda _0$	-	Base gene expression level, shared across all genes and cell types
$\Lambda _{gt}$	-	Overexpression for a marker gene *g* in its specific cell type *t*
$N_s$	Truncated normal	Number of cells in spot *s*
$p_g$	-	Success probability parameter for gene *g*
$Z_{st}$	Bernoulli	Indicator variable stating presence of cell type *t* in spot *s*
$\pi _{st}$	Beta	Prior for the probability of presence of cell type *t* in a spot *s*
Deterministic variables		
$h_{st}$	Dirichlet	Fraction of cell type *t* in a spot *s*
$\mu _{gs}$	-	Average expression of gene *g* in a spot *s*

**Table 2:** Description of hyperparameters of Celloscope’s variables

Hyperparameters	
$\alpha$	Adjustment for the average number of cell types present in a spot
*a*	Rate parameter for cell types present in a spot
*b*	Scale parameter for cell types present in a spot
$a_0$	Rate parameter for cell types absent in a spot
$b_0$	Scale parameter for cell types present in a spot
$l_s$	Estimate for the total number of cells in a spot
$\sigma$	Prior strength for $l_s$

The key assumption behind Celloscope is that marker genes are over-expressed in their specific cell types. To account for the increased expression of the marker genes in their specific cell types as compared to their base expression, we introduce the following two hidden variables: (i) $\Lambda _{gt}$ as an over-expression of gene *g* when it is a marker for type *t* and (ii) $\lambda _0$ as a base expression of any gene shared across all the genes (for cell types other than specific for *g*). Let us consider a cell *c* of type *t*. If gene *g* is a non-marker gene for cell type *t*, then its expression in cell *c* is equal to $\lambda _0$. However, if gene *g* is a maker gene for cell type *t*, then its expression in cell *c* is equal to $\lambda _0 + \Lambda _{gt}$.

Our main goal is to estimate the proportions of given cell types across all the spots, represented by the hidden variable *H*, which is a matrix with $s_M$ rows and $t_N$ columns. The value of an element $h_{st}$ is the proportion of all cells in spot *s* that are of type *t*, with values from 0 to 1. One row of the matrix *H*, denoted as $H_{s:} = [h_{st_1}, \dots , h_{st_N}]$ represents the hidden composition of spot *s*. Obviously, entries of a given row sum up to 1.

For a given gene *g*, its expression $C_{gs}$ is measured as the count of reads from spot *s* that map to gene *g*. The gene expression matrix *C* is modeled as an observed variable. A row $C_{g:} = [C_{gs_1}, \dots , C_{g {s_M} }]$ represents the expression profile for gene *g* across cell types and a column $C_{:s} = [C_{{g_1}s}, \dots , C_{ {g_K} s}]$ represents expression profile for a spot *s* across marker genes. We assume that the expected value of the random variable $C_{gs}$ depends on the hidden composition $H_{s:}$ of the cell types in spot *s*. In the subsequent discussion, we shall use the following three well-known remarks:

#### Remark 1

$X \sim NB(r, p)$ is equivalent to $X \sim NB\left( \frac{1-p}{p} \mu , p\right)$, where $\mu = \frac{p}{1 - p} r$.

#### Remark 2

Let *X*, *Y* be independent random variables satisfying $X \sim NB(r_1, p)$, $Y \sim NB(r_2,p)$. Then:

$$\begin{aligned} X + Y \sim NB(r_1+r_2, p). \end{aligned}$$

#### Remark 3

Let $X_1,\;\dots ,\;X_{k}$ be mutually independent random variables, $X_i \sim Gamma(\alpha _i, \beta ),\; i=1,\dots ,k$ and $Y_i=\frac{X_i}{X_1+\cdots +X_{k}}$. Then, the joint distribution satisfies $(Y_1, Y_2, \dots , Y_k) \sim Dirichlet(\alpha _1, \alpha _2, \dots , \alpha _k)$.

Let us consider a single cell of an unknown type *t* present at the spot *s*. We denote expression of gene *g* in this cell as $C_{gs}^{\text {single cell}}$. We use the negative binomial distribution to model gene expression. This distribution has two parameters: $r_{gt}$―the rate parameter dependent on the gene and cell type in question―and $p_g$―success probability dependent only on the gene in question. The variable $p_g$ enables us to take into account the over-dispersion in gene expression data. We assume that:

$$\begin{aligned} C_{gs}^{\text {single cell}} \sim NB \left( r_{gt}^{ \text {single cell} },\; p_g \right) . \end{aligned}$$

The average expression level of the gene *g* for that cell is equal to $\lambda _0 + B_{gt} \Lambda _{gt}$. Thanks to Remark 1, we can express the rate parameter $r_{gt}^{ \text {single cell} }$ as the scaled mean of the considered distribution and we obtain:

$$\begin{aligned} C_{gs}^{\text {single cell}} \mid B_{gt}, \Lambda _{gt}, \lambda _0, p_g \sim NB \left( \frac{1-p_g}{p_g} \left( \lambda _0 + B_{gt} \Lambda _{gt}\right) ,\; p_g \right) . \end{aligned}$$

Obviously, a given spot *s* contains more than only one cell. Let us assume that at a given spot *s* there are $n_{st}$ cells of type *t*. Then, we have

$$\begin{aligned} C_{gs} {\mathop {=}\limits ^{d}} n_{st_1} C_{gs}^{t_1} + n_{st_2} C_{gs}^{t_2} + ... + n_{st_n} C_{gs}^{t_n}, \end{aligned}$$

where ${\mathop {=}\limits ^{d}}$ denotes equality in distribution.

Remark 2 gives us:

$$\begin{aligned} C_{gs} \mid B_{g:}, \lambda _0, \Lambda _{g:}, p_g, n_{s:} \sim NB \left( \sum \limits _t n_{st} \frac{1-p_g}{p_g} (\lambda _0 + B_{gt} \Lambda _{gt}) ,\; p_g \right) . \end{aligned}$$

The total number of cells in spot *s* is represented by a hidden variable $N_s$. We use the truncated normal distribution as the prior on $N_s$ with mean $l_s$ and variance $\sigma$. While $l_s$ is estimated based on H&E image analysis, the latter accounts for prior strength and the level of our belief in confidence in the results of the number of cells estimation. Since $n_{st} = N_s h_{st}$, we have that:

$$\begin{aligned} C_{gs} \mid B_{g:}, \Lambda _{g:}, \lambda _0, p_g, N_s, H_{s:} \sim NB \left( N_s \frac{1-p_g}{p_g}\sum \limits _t h_{st} (\lambda _0 + B_{gt} \Lambda _{gt}) , \; p_g \right) . \end{aligned}$$

Denoting $\mu _{gs} = \sum \limits _t h_{st} (\lambda _0 + B_{gt} \Lambda _{gt})$, we obtain:

1 $$\begin{aligned} C_{gs} \mid \mu _{gs}, N_s, p_g \sim NB \left( N_s \frac{1-p_g}{p_g} \mu _{gs} , \; p_g \right) . \end{aligned}$$

We use a simple feature allocation model to represent the presence of cell types in spots. We set:

2 $$\begin{aligned} \pi _{st} \sim Beta \left( \frac{\alpha }{t_N}, 1 \right) . \end{aligned}$$

Let $Z_{st}$ indicate whether type *t* is present in spot *s* (takes value 1, if *t* is present in *s* and value 0 otherwise). We set:

3 $$\begin{aligned} Z_{st} \sim Bernoulli(1, \pi _{st}). \end{aligned}$$

Let $\theta _{st}$ denote the unnormalized abundance of type *t* in spot *s*. In the case when a given type *t* is present in spot *s* ($Z_{st}=1$), we expect $\theta _{st}$ to take values more distant from 0 as compared to a situation when $Z=0$. Therefore, we define a conditional distribution for $\theta _{st}$ given the value of $Z_{st}$ in the following manner:

4 $$\begin{aligned} \theta _{st} \mid Z_{st}= &\; {} 1 \sim Gamma(a,b)\nonumber \\ \theta _{st} \mid Z_{st}= &\; {} 0 \sim Gamma(a_0,b_0) \end{aligned}$$

where $a,b,a_0,b_0$ are chosen so that values sampled from *Gamma*(*a*, *b*) are significantly larger than small values sampled from $Gamma(a_0,b_0)$. Let us denote $\Theta _{s:} = [\theta _{st_1}, \theta _{s_{t_2}}, \dots , \theta _{s{t_{N}}}]$ as one row of matrix $\Theta$ describes unnormalized abundance of all cell types in spot *s*.

The proportion $h_{st}$ of cell type *t* in spot *s* is deterministically computed based on inferred $\Theta _{s:}$. For a fixed spot *s*:

5 $$\begin{aligned} h_{st} = \frac{{\theta }_{st}}{\sum \limits _{t = 1}^{t_N} {\theta }_{st}}. \end{aligned}$$

Remark 3 gives the conditional probability distribution of $H_{s:}$ given $\Theta _{s:}$

$$\begin{aligned} H_{s:} | \Theta _{s:} \sim Dirichlet(\theta _{s{t_1}}, \theta _{s_{t_2}}, \dots , \theta _{st_{N}}). \end{aligned}$$

### Celloscope’s parameters inference

For inferring the hidden variables we use the Metropolis-within-Gibbs sampler, a Markov chain Monte Carlo (MCMC) algorithm which is a combination of the Gibbs Sampler and the Metropolis-Hastings algorithm (Algorithm 1). Suppose the graphical model contains variables $x_1, \dots , x_n$. Let $MB(x_i)$ denote the Markov Blanket of $x_i$, i.e., the set containing its parents, children, and co-parents. Then:

6 $$\begin{aligned} P(x_i \mid x_1, \dots , x_{i-1}, x_{i+1},\dots , x_n) = P \left( x_i \mid \text {MB}(x_i)\right) , \end{aligned}$$

i.e., the conditional distribution of $x_i$ given the values of all other variables equals the conditional distribution given the values of the variables from its Markov blanket.

The iterative sampling procedure is as follows: firstly, starting values for $x_1^{(0)},\dots , x_n^{(0)}$ are randomly initialized, then, in a given iteration *j* ($j = 1, 2, \dots , J$, where *J* denotes the number of iterations), we take every variable $x_i$, $i=1, ..., n$ one by one, in some arbitrary ordering and for each given variable $x_i$, its value is sampled given the values of the variables from the Markov blanket $\text {MB}\Big (x_i^{(j-1)}\Big )$ from the previous iteration. As a result, each $x_i$ is updated iteratively, up until convergence. There are two options for updating the value of $x_i$ in the *j*th iteration:

1. If $P\left( x_i \mid \text {MB}\left( x_i^{(j-1)}\right) \right)$ can be expressed in a closed form, a new value $x^*_i$ is sampled directly from $P\Big (x_i \mid \text {MB}\left( x_i^{(j-1)}\right) \Big )$ and $x_i^{(j)}$ is set to $x^*_i$.
2. In case we only know a function *f* proportional to $P\left( x_i \mid \text {MB}(x_i)\right)$
  $$\begin{aligned} P \left( x_i \mid MB \left( x_i^{(j-1)}\right) \right) \propto f(x_i), \end{aligned}$$
  we perform a single Metropolis-Hastings accept-reject step (MH-single-step procedure in Algorithm 1). A candidate value $x_i^*$ is sampled from a predefined proposal distribution $q \left( \cdot \mid x^{(j-1)}_i\right)$, and then either accepted with probability given by
  7 $$\begin{aligned} r = \min \left( 1, \frac{f\left( x^* \right) q\left( x^{(j-1)} \mid x^* \right) }{f\left( x^{(j-1)}\right) q\left( x^* \mid x^{(j-1)}\right) } \right) \end{aligned}$$
  and $x^{(j)}_i \leftarrow x_i^*$, or the previous value is held: $x^{(j)}_i \leftarrow x_i^{(j-1)}$. After updating $x_i$, we immediately use the new value for sampling other variables.

**Figure:**
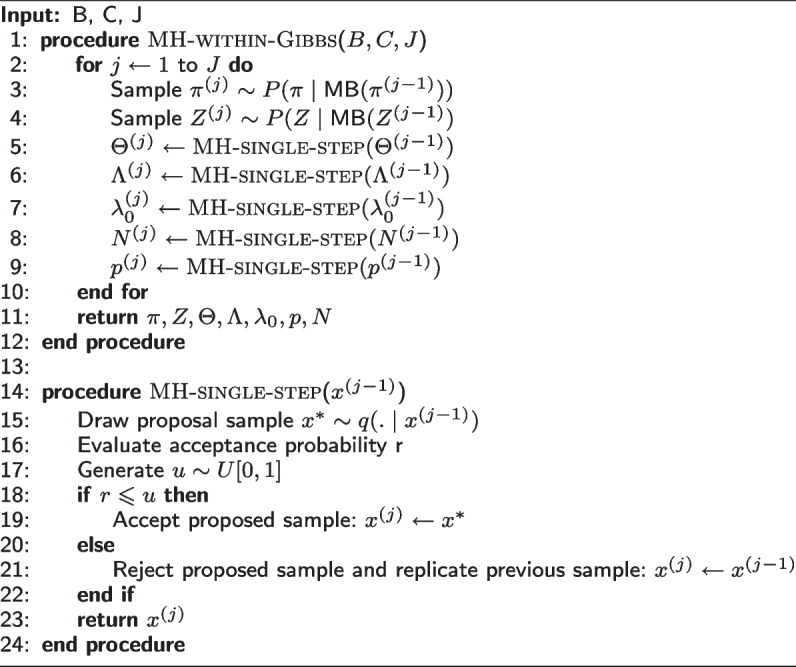
**Algorithm 1** Metropolis-Hastings within Gibbs Sampler of Celloscope’s parameters

Below, we provide the model’s equations for target distributions, for which, in the case of the first group of variables, namely $\pi$ and *Z*, we know the explicit formulas. In the case of the second group of variables: $\Theta$, $\Lambda$, $\lambda _0$, *p*, *N,* only functions $f(\cdot )$ proportional to the target distributions are known. These functions are used to compute the acceptance ratios (Eq. 7), while performing single accept-reject Metropolis-Hastings steps.

#### Sampling $\pi _{st}$

 

8 $$\begin{aligned} P\left( \pi _{st} \mid Z_{st}\right) \propto \textrm{Beta} \left( \pi _{st} \mid \frac{\alpha }{t_N}, 1 \right) \textrm{Bernoulli}\left( Z_{st} \mid 1, \pi _{st}\right) = \textrm{Beta} \left( \pi _{st} \mid \frac{\alpha }{t_N}+ Z_{st}, 2 - Z_{st} \right) \end{aligned}$$

#### Sampling $Z_{st}$

As $Z_{st}$ is a discrete, binary random variable, it suffices to consider its two possible values, 0 and 1.

9 $$\begin{aligned}&P(Z_{st} \mid \theta _{st}, \pi _{st}) \propto P(Z_{st} \mid \pi _{st}) P(\theta _{st} \mid Z_{st}) = \textrm{Bernoulli}(Z_{st} \mid 1, \pi _{st}) \Gamma (\theta _{st} \mid a, b )^{Z_{st}} \Gamma (\theta _{st} \mid a_0, b_0 )^{1-Z_{st}}&\nonumber \\&P(Z_{st}=1 \mid \pi _{st}, \theta _{st}) \propto \pi _{st} \theta _{st}^{a-1} e^{-b \theta _{st}} = A&\nonumber \\&P(Z_{st}=0 \mid \pi _{st}, \theta _{st}) \propto (1 -\pi _{st}) \theta _{st}^{a_{0}-1} e^{- b_{0} \theta _{st}} = B&\nonumber \\&P(Z_{st} = 1 \mid \pi _{st}, \theta _{st}) = \frac{A}{A+B} \quad P(Z_{st} = 0 \mid \pi _{st}, \theta _{st}) = 1- \frac{A}{A+B} = \frac{B}{A+B}&\end{aligned}$$

#### Target distribution for unnormalized cell type abundance $\Theta _{s:}$

We update each spot independently. For a selected spot *s*:

10 $$\begin{aligned}{} & {} P\Big ( \Theta _{s:} \mid C_{:s}, B, Z_{s:}, \lambda _0, \Lambda , N_s, p \Big ) \propto \prod \limits _{g \in \text {Genes}} P \Big ( C_{gs} \mid \Theta _{s:}, B_{g:}, \Lambda _{g:}, \lambda _0, N_s, p_g\Big ) \prod \limits _{t \in \text {Types}} P(\theta _{st} \mid Z_{st})\nonumber \\{} & {} \qquad = \prod \limits _{g \in \text {Genes} } \textrm{NB} \Big ( C_{gs} \mid N_s \frac{1-p_g}{p_g} \mu _{gs} , \; p_g\Big ) \prod \limits _{t \in \text {Types}:\; Z_t \ne 0} \Gamma (\theta _{st} \mid a,b) \prod \limits _{t \in \text {Types}:\; Z_t = 0} \Gamma (\theta _{st} \mid a_{0}, b_{0}) \end{aligned}$$

#### Target distribution for $N_s$

For a selected spot *s*:

11 $$\begin{aligned}&P(N_s \mid C_{:s}, B, \Theta _{s:}, \lambda _0, \Lambda , p) \propto \prod _{g \in \text {Genes}} P(C_{gs} \mid \Theta _{s:}, B_{g:}, \Lambda _{g:},\lambda _0, N_s, p_g) P(N_s \mid l_s, \sigma ^2) =&\nonumber \\&\prod _{g \in \text {Genes}} \textrm{NB} \left( C_{gs} \mid N_s \frac{1-p_g}{p_g} \mu _{gs}, p_g \right) \textrm{TN}( N_s \mid l_s, \sigma )&\end{aligned}$$

#### Target distribution for $\lambda _0$

 

12 $$\begin{aligned}&P(\lambda _0 \mid C, B, \Theta , N, \Lambda , p) \propto \prod \limits _{\begin{array}{c} g \in \text {Genes} \\ s \in \text {Spots} \end{array}} P(C_{gs} \mid \Theta _{s:}, B_{g:}, \Lambda _{g:}, \lambda _0, p_g, N_s) =&\nonumber \\&\prod \limits _{\begin{array}{c} g \in \text {Gens} \\ s \in \text {Spots} \end{array}} \textrm{NB} \left( C_{gs} \mid N_s \frac{1-p_g}{p_g} \mu _{gs}, p_g \right)&\end{aligned}$$

#### Target distribution for $\Lambda _{gt}$

For a selected gene *g*:

13 $$\begin{aligned}&P \left( \Lambda _{g:} \mid C_{g:}, B_{g:}, \Theta , \lambda _0, N_s, p_g \right) \propto \prod \limits _{s \in \text {Spots} }P \Big ( C_{gs} \mid B, \Theta _{s:}, \Lambda _{g:}, \lambda _0, N_s, p_g \Big ) =&\nonumber \\&\prod \limits _{s \in \text {Spots} } \textrm{NB} \Big ( C_{gs} \mid N_s \frac{1-p_g}{p_g} \mu _{gs}, p_g \Big )&\end{aligned}$$

#### Target distribution for $p_g$

For a selected gene *g*:

14 $$\begin{aligned}&P\Big ( p_g \mid C_{g:}, B_{g:}, \Theta , \Lambda _{g:}, \lambda _0, N_s \Big ) \propto \prod \limits _{s \in \text {Spots} }P \Big ( C_{gs} \mid \Theta _{s:}, B_{g:}, \Lambda _{g:}, \lambda _0, N_s, p_g \Big ) =&\nonumber \\&\prod \limits _{s \in \text {Spots} } \textrm{NB} \Big ( C_{gs} \mid N_s \frac{1-p_g}{p_g} \mu _{gs}, p_g \Big )&\end{aligned}$$

#### Proposal distribution

Let $\Phi (x)$ denote the cumulative distribution function of *N*(0, 1), evaluated in point *x* and *q*(*y*|*x*) the proposal distribution, i.e., the conditional probability of proposing a new state *y* given the previous value was equal to *x*. We choose the truncated normal distribution $TN(\mu , \sigma )$ for the proposal distribution, since it allows for controlling the step size and it is appropriate for proposing values for non-negative variables. Note that for the truncated normal distribution we have that:

$$\begin{aligned} q(y|x) = \frac{1}{C} \frac{1}{\sqrt{2\pi } \sigma } \exp {\Big ( - \frac{(y-x)^2}{2\sigma ^2} \Big )}, \quad y>0, \end{aligned}$$

where $C = \Phi \Big (\frac{x}{\sigma } \Big )$ is a normalizing constant. Bearing this in mind, we compute the Hastings ratio:

15 $$\begin{aligned} \frac{q(x|y)}{q(y|x)} = \frac{ \frac{1}{\sigma \sqrt{2} \pi } \exp \Big{(} {- \frac{(x-y)^2}{2 \sigma ^2} } \Big{)} \frac{1}{C_1} }{ \frac{1}{\sigma \sqrt{2} \pi } \exp \Big{(} {- \frac{(y-x)^2}{2 \sigma ^2} } \Big{)} \frac{1}{C_2} } = \frac{C_2}{C_1} = \frac{\Phi \Big (\frac{x}{\sigma } \Big )}{\Phi \Big (\frac{y}{\sigma } \Big )}. \end{aligned}$$

Eq. (15) is used for all the model variables, except for $N_s$, which unlike the rest, takes integer values. Therefore, we choose the ceiling of the truncated normal distribution as the proposal distribution. To compute the Hastings ratio in this case, we first need to find the density of a random variable

$$\begin{aligned} Y = \lceil X \rceil , \text { where } X \sim TN(\mu , \sigma ), \end{aligned}$$

denoting the ceiling of a truncated normal random variable.

##### Remark 4

Let $\Phi _{TN}(x)$ denote the cumulative distribution function of $TN(\mu ,\sigma ^2)$ in point *x*. Then:

$$\begin{aligned} P(Y=x) = P \big ( \lceil X \rceil = x \big ) = \Phi _{TN}(x) - \Phi _{TN}(x-1), \text { where } \Phi _{TN}(x) = 1 - \frac{1 - \Phi \big (\frac{x-\mu }{\sigma } \big )}{\Phi \big (\frac{\mu }{\sigma } \big )}. \end{aligned}$$

##### Proof

$$\begin{aligned} P(Y>k)= & {} P(\lceil X \rceil> k) = P(X>k) = 1 - \Phi _{TN}(k) \\ P(Y=k)= & {} P(Y>k-1) - P(Y>k) = \Phi _{TN}(k) - \Phi _{TN}(k-1). \end{aligned}$$

$$\square$$

Now we can compute the Hastings ratio used for $N_s$:

$$\begin{aligned} \frac{q(x|y)}{q(y|x)} = \frac{\Phi _{TN(y,\sigma ^2)}(x) - \Phi _{TN(y, \sigma ^2)}(x-1) }{\Phi _{TN(x,\sigma ^2)}(y) - \Phi _{TN(x,\sigma ^2)}(y-1) } = \frac{ \Phi \Big (\frac{x}{\sigma } \Big ) }{ \Phi \Big (\frac{y}{\sigma } \Big )} \cdot \frac{ \Phi \Big ( \frac{x-y}{\sigma } \Big ) - \Phi \Big ( \frac{x-1-y}{\sigma } \Big ) }{ \Phi \Big ( \frac{x-y}{\sigma } \Big ) - \Phi \Big ( \frac{y-1 -x}{\sigma } \Big ) } . \end{aligned}$$

#### The dummy type

In order to account for cell types that potentially exist in the examined tissue but are unknown or not considered and thus are not represented in the cell type marker matrix *B*, we introduce a so-called *dummy type*. We assume that the set of dummy type’s marker genes is separate from the set of all markers of the modeled cell types. Therefore, technically, to model the dummy type, we insert an additional column *t* for the dummy type and fill it with zeros, so that for all $g \in \text {Genes}$ we have $B_{gt} = 0$.

#### Adaptive step size

Let us recall that we employ the truncated normal distribution $TN(\cdot , \sigma )$ to propose a new value in the Metropolis-Hastings accept-reject step in Algorithm 1. The variance parameter $\sigma$ corresponds to the *step size* of the sampler and affects the speed of convergence to the target distribution. To accelerate the convergence, we modify the algorithm in such a way that we use a different step size for every sampled variable and adapt it as the sampling procedure progresses, aiming at achieving the optimal acceptance ratio of 23% [61]. Specifically, for a selected model’s variable, we start with an arbitrary value for its step size, and once in every 10,000 iterations in the case of mouse brain data and 50,000 in the case of the human prostate data, we modify the step size according to the changing acceptance ratio. If the acceptance ratio is smaller than the optimal acceptance ratio, the step size is modified according to $\sigma \leftarrow (1-\epsilon ) \sigma$, otherwise $\sigma \leftarrow (1+\epsilon ) \sigma$, where $\epsilon$ denotes a parameter controlling the strength of modification. Importantly, this procedure is restricted to the burn-in phase.

### Synthetic data simulation scenarios

We considered four data simulating scenarios that differ with respect to the average number of distinct cell types present in each spot and the way the number of cells in each spot is accounted for in the model. In the first case, we either assume an increased number of cell types (dense scenario) or a decreased number of cell types (sparse scenario). While the dense scenario on average resulted in circa five cell types present in each spot, the sparse scenario resulted in circa two different cell types in each spot. When it comes to the second aspect, the way the number of cells in each spot is accounted for in the model; in the first option, we use the number of cells in each spot in the inference as the model’s input (as known values), i.e, $N_s$ variables become observed and their values are fixed to the true values. In the second option, we incorporate them as values for $l_s$ hyperparameters of the $N_s$ variables (as, so-called, priors), i.e., the $N_s$ are hidden, inferred variables, and their prior mean is fixed to the true values.

### Synthetic data simulation

We set $s_M=800$, $t_N=7$, $a=10,\; b=1,\; a_0=0.1,\; b_0=1,\; \lambda _0=0.2$ and the number of marker genes $g_K=149$ (marker genes distributed across cell types as: 15, 31, 35, 23, 17, 33, 0). The value of the parameter $\alpha$ depends on the data simulating scenario, i.e. for the dense scenario we fix $\alpha = 2t_N$ and for the sparse scenario $\alpha = 0.45t_N$. To sample values for gene expression level $\Lambda$, we first calculate the average gene expression for cell types found in a scRNA-seq dataset on the mouse brain cortex [62]. The obtained values were resampled to finally get values of $\Lambda$ for all marker genes. The values for $p_g$ were sampled from *Unif*(0, 1), $\pi$ according to the distribution (2), *Z* according to distribution (3) and $\Theta$ according to (4). We computed *H* based on $\Theta$, from Eq. (5).

After all values for hyperparameters have been established, for each of the four considered scenarios, 15 replicates were sampled from the generative model Eq. (1). As a result, we obtained 60 synthetic datasets.

### Quality measure of the inference accuracy in synthetic data

For each of 60 synthetic datasets, we calculate:

16 $$\begin{aligned} \epsilon _{k} = \frac{ \sum \limits _{i=1}^{s_M} \sum \limits _{j=1}^{t_N } |h_{ij}^{(k)} - \hat{h}_{ij}^{(k)}|}{t_N s_M}, \end{aligned}$$

where $\epsilon _k$ denotes the average error across spots and cell types for the *k*th datasets, $k=1, \dots , 60$, $h_{ij}^{(k)}$ denotes the true value of a proportion of a type *j* in a spot *i* for the *k*th dataset and $\hat{h}_{ij}^{(k)}$ is the proportion of a type *j* in a spot *i* estimated by Celloscope for the *k*th dataset, Stereoscope or RCTD.

### Run settings for mouse brain and human prostate data

In the following, we describe Celloscope’s run settings for the mouse brain dataset sagittal section, mouse brain data coronal section in curly brackets and provide the corresponding settings used for the human prostate data in parentheses.

We ran three (ten) independent chains with the same hyperparameters (Table 3), but random starting values with 120,000 ($10^6$) {40000} total iterations, including the burn-in phase of the first 90,000 (900,000) {30000} iterations. The estimated total number of cells in each spot ($l_s$) was incorporated as priors for the $N_s$ variable. For $\Theta , \Lambda , \lambda _0, p_g$, we introduced adaptive step sizes (with the optimal acceptance ratio of 23%). Table 4 presents the starting values for the step sizes. Additionally, we set the thinning parameter to 10 (100), which resulted in keeping every 10th (100th) value and discarding the rest of them. Additionally, to impose shrinkage prior on the proportion of the *dummy type*, we manually set $Z_{: t}=0$, where *t* here denotes *the dummy type*. Finally, estimates obtained via independent chains were averaged.

**Table 3:** Values of hyperparameters used for mouse brain data sagittal section, if different for mouse brain coronal section {in curly bracket} and human prostate data (in parentheses)

Variable	Hyperparametrs
$\theta$	*a* = 10,$b=1$,$a_0=0.1$,$b_0=1$
$\pi$	$\alpha =10$(12) {8}
$\sigma$	4 (5) {3}

**Table 4:** Step sizes’ values for proposal proposal distributions used to update variables with a single accept-reject Metropolis-Hastings step.$*$denotes adaptive step size

Variable	Step size
$\theta$	0.1
$N_s$	2.01
$\lambda _0$	$0.05^{*}$
$p_g$	$0.1^{*}$

### Recommendation on parameters choice on new data sets

#### Cell counting

Default parameters values for the cell counting procedure are provided in the script available at GitHub (https://github.com/szczurek-lab/qupath-spot-utils). Due to the fact that the results of the estimation can be validated on the respective H&E images in QuPath for a representative, a limited number of spots, the parameters given to the algorithm can be tuned manually based on the user’s visual inspection. Importantly, QuPath inbuilt cell counting algorithm is guided by numerous parameters, e.g., setting too high *sigma* (which is responsible for smoothing) may result in blurring some nuclei together. Moreover, while on the one hand a too high *threshold* parameter may result in too few pixels passing it and lowered sensitivity of the cell counting algorithm, on the other hand, a too low *threshold* value may result in many false positives. For more information on the cell counting procedure and detailed guidance on parameters choice, see QuPath tutorial [63, 64].

#### Running Celloscope

The recommended default values for Celloscope’s parameters are given in Table 5.

**Table 5:** Default values of Celloscope’s parameters

Parameter	Default value
Step-size$\theta$	0.1
Step-size$\lambda _0$	0.05
Step-size$p_g$	0.1
Step-size$N_s$	2
(a, b,$a_0$,$b_0$)	(10, 1, 0.1, 1)
$\alpha$	$> \frac{t_N}{t_N+1}$
Burn-in	$\ge 40,000$
Number of iterations	$\ge 50,000$
Thinning	10

The parameter corresponding to the strength of the prior distribution over the total number of cells ($\sigma$) in each ST spot should reflect our confidence in the accuracy of the results of the preliminary estimation based on H&E slides performed with the cell counting procedure.

We recommend a data-driven approach to set the initial values for the $\Lambda$ parameter matrix and its step-sizes. On default, for each gene, Celloscope takes as initial values for $\Lambda _{g\cdot }$ an average of non-zero entries of $C_{g\cdot }$, and half of those values as step-sizes for $\Lambda$ parameters.

The parameter $\alpha$ controls the feature allocation model that governs the expected number *D* of different cell types expected to be present in each spot:

$$\begin{aligned} D = \mathbb {E} \left[ \sum _t Z_{st}\right] = t_N \frac{ \frac{\alpha }{t_N} }{1+\frac{\alpha }{t_N}} = \frac{N \alpha }{N + \alpha } \Rightarrow \alpha = \frac{D t_N}{t_N - D},\; D<t_N. \end{aligned}$$

Therefore, we can choose $\alpha$, so that *D* reflects our intuitions about the number of different cell types that an average spot contains.

As for all MCMC methods, decisions about the number of the burn-in iterations should be based on some convergence diagnostics, such as Gelman-Rubin [65] or Geweke [66] statistics. However, we additionally advise to diagnose convergence with the use of trace plots. Due to the high number of hidden variables and parameters in the model, we suggest analyzing the proportions of cell types for a small, randomly selected number of spots (Additional file 1: Fig. S11). Lastly, by default, the step-sizes are updated once in every 10,000 iterations. In case of a lower number of all iterations, this parameter can be slightly lowered.

### Supplementary Information

**Additional file 1: Supplementary Information.** Additional file 1 contains Supplementary Text and Supplementary Figures.

**Additional file 2: Table S1.** Marker genes for the mouse brain dataset.

**Additional file 3: Table S2.** Marker genes for the mouse brain dataset.

**Additional file 4: Table S3.** Marker genes for the human prostate dataset.

**Additional file 5.** Review history.

## Data Availability

Celloscope implementation is freely accessible under a GNU General Public License v3.0 license at GitHub [67] and Zenodo [68]. The cell counting procedure is available at GitHub [69] and Zenodo [70] under a GNU General Public License v3.0 license. The results were plotted using ggplot2 [71]. Count matrices for human prostate data are available at https://www.spatialresearch.org/resources-published-datasets [20]. Sequencing data are deposited at the European Genome-Phenome Archive (EGA), hosted by the European Bioinformatics Institute (EBI), under the accession number EGAS00001003001 [72]. Mouse brain sagittal section data is available at https://support.10xgenomics.com/spatial-gene-expression/datasets/1.1.0/V1_Mouse_Brain_Sagittal_Anterior and was accessed via SeuratData R package (v.0.1.1) [50, 51]. Mouse brain coronal section data is available at https://support.10xgenomics.com/spatial-gene-expression/datasets/1.1.0/V1_Adult_Mouse_Brain and was downloaded from [13, 73]. Simulated data are available at GitHub [67] and Zenodo [68]. Real data used to obtain results presented in the manuscript are available at GitHub [67] and Zenodo [68]. For each dataset, we deposited gene expression matrix, prior knowledge on marker genes, and the results of the cell counting procedure. In case of human prostate data, we deposited the subset of spots annotated as an inflammation region and analyzed in this manuscript.
