# Supplementary material for: Celloscope: a probabilistic model for marker-gene-driven cell type deconvolution in spatial transcriptomics data
Source: Genome Biol. 2023 May 17;24:120. doi: 10.1186/s13059-023-02951-8 (PMC10190053; doi:10.1186/s13059-023-02951-8)
Supplement: Supplementary file 1 — Additional file 1: Supplementary Information. Additional file 1 contains Supplementary Text and Supplementary Figures. [file 13059_2023_2951_MOESM1_ESM.pdf]

Celloscope: a probabilistic model for cell type deconvolution in spatial transcriptomics data Supplementary Information

Agnieszka Geras[1,2], Shadi Darvish Shafigh[2,3], Kacper Domżał[2], Igor Filipiuk[2], Alicja Rączkowska[2], Paulina Szymczak[2], Hosein Toosi[4], Leszek Kaczmarek[5], Łukasz Koperski[6], Jens Lagergren[4], Dominika Nowis[7], Ewa Szczurek[2*]

[1]Faculty of Mathematics and Information Science, Warsaw University of Technology, Warsaw, Poland, [2]Faculty of Mathematics, Informatics, and Mechanics, University of Warsaw, Warsaw, Poland, [3]Sorbonne Université, CNRS, IBPS, Laboratoire de Biologie, Computationnelle et Quantitative - UMR, Paris, France, [4]Royal Institute of Technology, Stockholm, Sweden, [5]BRAINCITY, Nencki Institute of Experimental Biology of the Polish Academy of Sciences, Warsaw, Poland, [6]Department of Pathology, Medical University of Warsaw, Warsaw, Poland, [7]Laboratory of Experimental Medicine, Medical University of Warsaw, Warsaw, Poland, [*] szczurek@mimuw.edu.pl

Supplementary Text

S1 Running Stereoscope

We ran Stereoscope using the following command:

```
stereoscope run --sc_fit true_gene_expression.tsv p_g.tsv --st_cnt C_gs.tsv -ste 75000
    -stb 256 -lr 0.01 --gpu -o.
```

`true_gene_expression.tsv` contains true values for gene expression profiles for each gene in each cell type. `p_g.tsv` contains true values for the over-dispersion parameter p_g.

S2 Running RCTD

We ran RCTD using the following commands:

```
nUMI <- colSums(counts)
SpatialRNA_instance <- SpatialRNA(coords, counts, nUMI)
myRCTD <- create.RCTD(SpatialRNA_instance, reference, max_cores = 7, gene_cutoff = 0,
    fc_cutoff = 0, gene_cutoff_reg = 0, fc_cutoff_reg = 0)
myRCTD@cell_type_info$info[[1]] <- true_gene_expression
bulk = fitBulk(myRCTD)
sigma = choose_sigma_c(bulk)
my_results <- fitPixels(sigma, doublet_mode = 'full')
norm_weights = sweep(my_results@results$weights, 1,
    rowSums(my_results@results$weights), '/')
```

`counts` denotes ST counts matrix and `coord` denotes spots coordinates. Parameters: gene_curoff and fc_cutoff, hene_cutodd_reg and cutoff_reg were all set to 0, so that all marker genes were used for further cell type deconvolution. Since the mean expression was not calculated from scRNAseq data, but rather had to be given to the model directly, `cell_type_info$info[[1]]` was set to `true_gene_expression`, which consisted of true values for gene expression level for each gene in each cell type. This enables to perform the process of cell type deconvolution and cell type normalization without a reference scRNA-seq data set.

S3 Running SpatialDWLS

We ran SpatialDWLS using the following commands:

```
sc_matrix<-read.csv("true_gene_expression.csv", row.names = 1, header= TRUE)
# data frame with col containing vector with single cell labels
sc_lable = as.data.frame(colnames(read.csv("true_gene_expression.csv", row.names =
    1, header= TRUE)))
colnames(sc_lable) = 'single_cell_labels'

sc_cortex <- createGiottoObject(raw_exprs = sc_matrix, instructions = instrs)
sc_cortex <- normalizeGiotto(gobject = sc_cortex)
sc_cortex@cell_metadata$leiden_clus<-as.character(sc_lable$single_cell_labels)
Sig = as.matrix(read.csv("true_gene_expression.csv", row.names = 1, header= TRUE))

grid_exp = read.csv("C_gs.csv", row.names = 1, header= TRUE)
grid_seqFish <- createGiottoObject(raw_exprs = grid_exp, instructions = instrs)
grid_seqFish <- normalizeGiotto(gobject = grid_seqFish)
grid_seqFish <- calculateHVG(gobject = grid_seqFish)
gene_metadata = fDataDT(grid_seqFish)
gene_metadata$hvg = 'yes'

featgenes = gene_metadata[hvg == 'yes']$gene_ID
grid_seqFish <- runPCA(gobject = grid_seqFish, genes_to_use = featgenes,
    scale_unit = F)
grid_seqFish <- createNearestNetwork(gobject = grid_seqFish, dimensions_to_use =
    1:10, k = length(colnames(sc_matrix)))
grid_seqFish <- doLeidenCluster(gobject = grid_seqFish, resolution = 0.4,
    n_iterations = 1000)
grid_seqFish<-runDWLSDeconv(gobject = grid_seqFish, sign_matrix = Sig)

res_df = grid_seqFish@spatial_enrichment$DWLS
rownames(res_df) = res_df$cell_ID
res_df = res_df[,-c(1)]
```

Here `true_gene_expression` serves as a simulated scRNAseq dataset in which one cell type is represented by one cell. We use single cell labels as leiden clusters as in the SpatialDWLS tutorial (`https://github.com/rdong08/spatialDWLS_dataset/blob/main/codes/seqFISH_plus_deconvolution.Rmd`).

S4 Running CellAssign

We ran CellAssign using the following commands:

```
calculated_sum_factors = calculateSumFactors(C_gs)
fit <- cellassign(exprs_obj = C_gs, marker_gene_info = B,
    s = calculated_sum_factors, learning_rate = 10^(-2), shrinkage = TRUE).
```

`C_gs` denotes gene expression matrix. `B` denotes a binary matrix with prior knowledge on marker genes. Learning rate was set to 10^{-1} for mouse brain and human prostate data and to 10^{-3} in the case of simulated data.

S5 Running BayesPrism

We ran BayesPrism using the following commands:

```
c_gs <- t(read.csv("C_gs.csv", row.names = 1, header= TRUE))
true_gene_expression <- t(read.csv("true_gene_expression.csv", row.names = 1, header=
    TRUE))
cell.type.labels = rownames(true_gene_expression)
cell.state.labels = rownames(true_gene_expression)
myPrism <- new.prism(
  reference= true_gene_expression,
  mixture = c_gs,
  input.type = "GEP",
  cell.type.labels = cell.type.labels,
  cell.state.labels = cell.state.labels,
  key = NULL,
  outlier.fraction=1.)
bp.res <- run.prism(prism = myPrism, n.cores=40)
theta <- get.fraction (bp=bp.res,
           which.theta="final",
           state.or.type="type")
```

We run BayesPrism using the GEP (gene expression profiles) mode. We used `true_gene_expression` that contains one scRNAseq expression observation for each cell type. While constructing the object `new.prism`, we set the key parameter (used to inform the model, which cells come from cancer tissue) to `NULL`, so that BayesPrism treats every cell type equally.

S6 Running STdeconvolve

We applied STdeconvolve to the coronal and sagittal mouse brain data and human prostate cancer data with the following commands:

```
cd <- as.matrix(read.csv("ST_data.csv", row.names = 1, header= TRUE))
counts <- cleanCounts(cd, min.lib.size = 100)
corpus <- restrictCorpus(counts, removeAbove=1.0, removeBelow = 0.05)
ldas <- fitLDA(t(as.matrix(corpus)), Ks = seq(2, 30, by = 1),
    ncores=parallel::detectCores())
optLDA <- optimalModel(models = ldas, opt = "min")
results <- getBetaTheta(optLDA)
deconProp <- results$theta
deconGexp <- results$beta
```

We set the maximal value of **Ks** parameter (the maximal number of cell types expected in data) to 30.

S7 Accessing mouse brain data

The count matrix was accessed via `SeuratData` [50] with the following commands:

```
InstallData("stxBrain")
brain <- LoadData("stxBrain", type = "anterior1")
```

More information: `https://satijalab.org/seurat/v3.2/spatial_vignette.html`.

Supplementary Figures

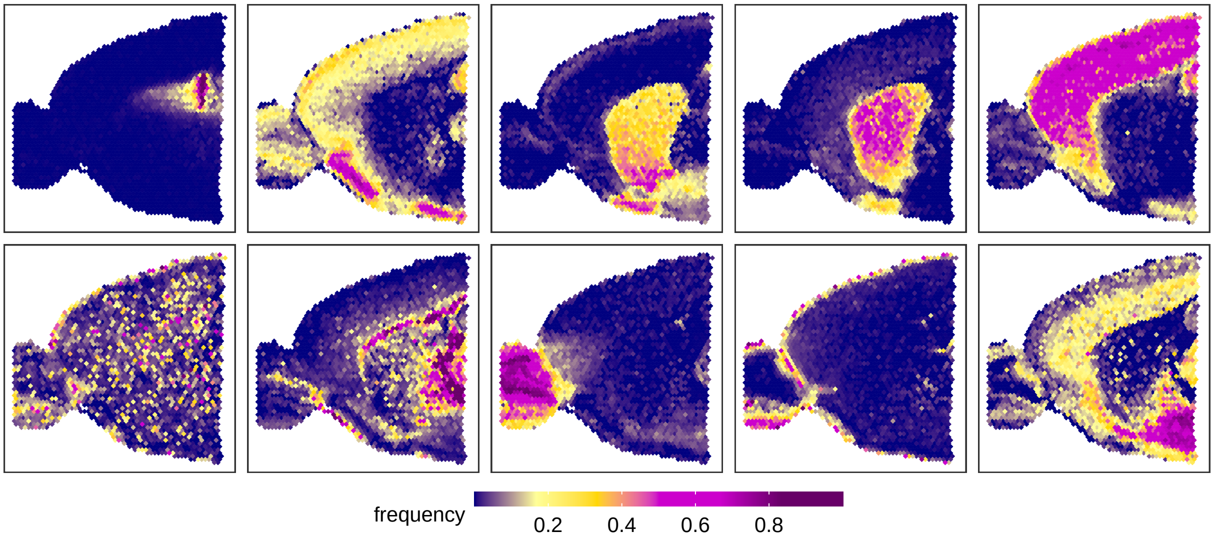

Figure S1: Results obtained with STdeconvolve for mouse brain data (sagittal section).

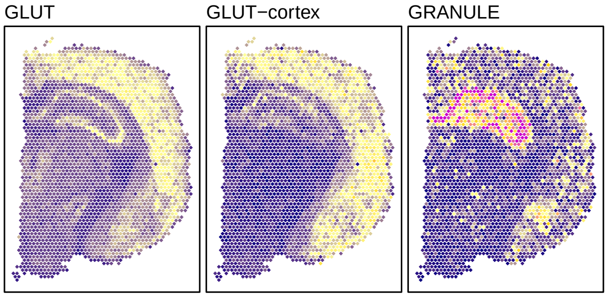

Figure S2: Glutamatergic neurons indicated in mouse brain data coronal section.

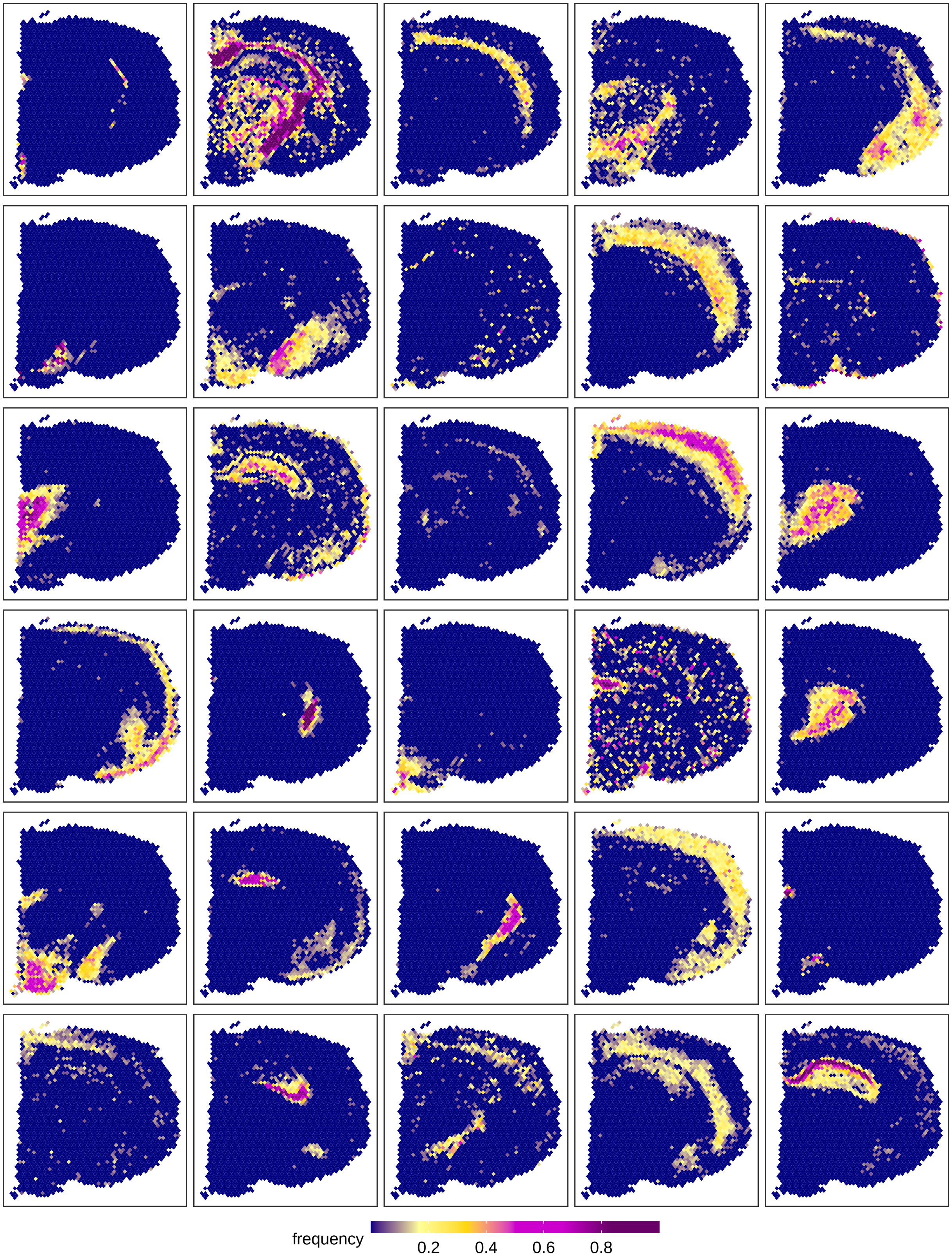

Figure S3: Results obtained with STdeconvolve for mouse brain data (coronal section).

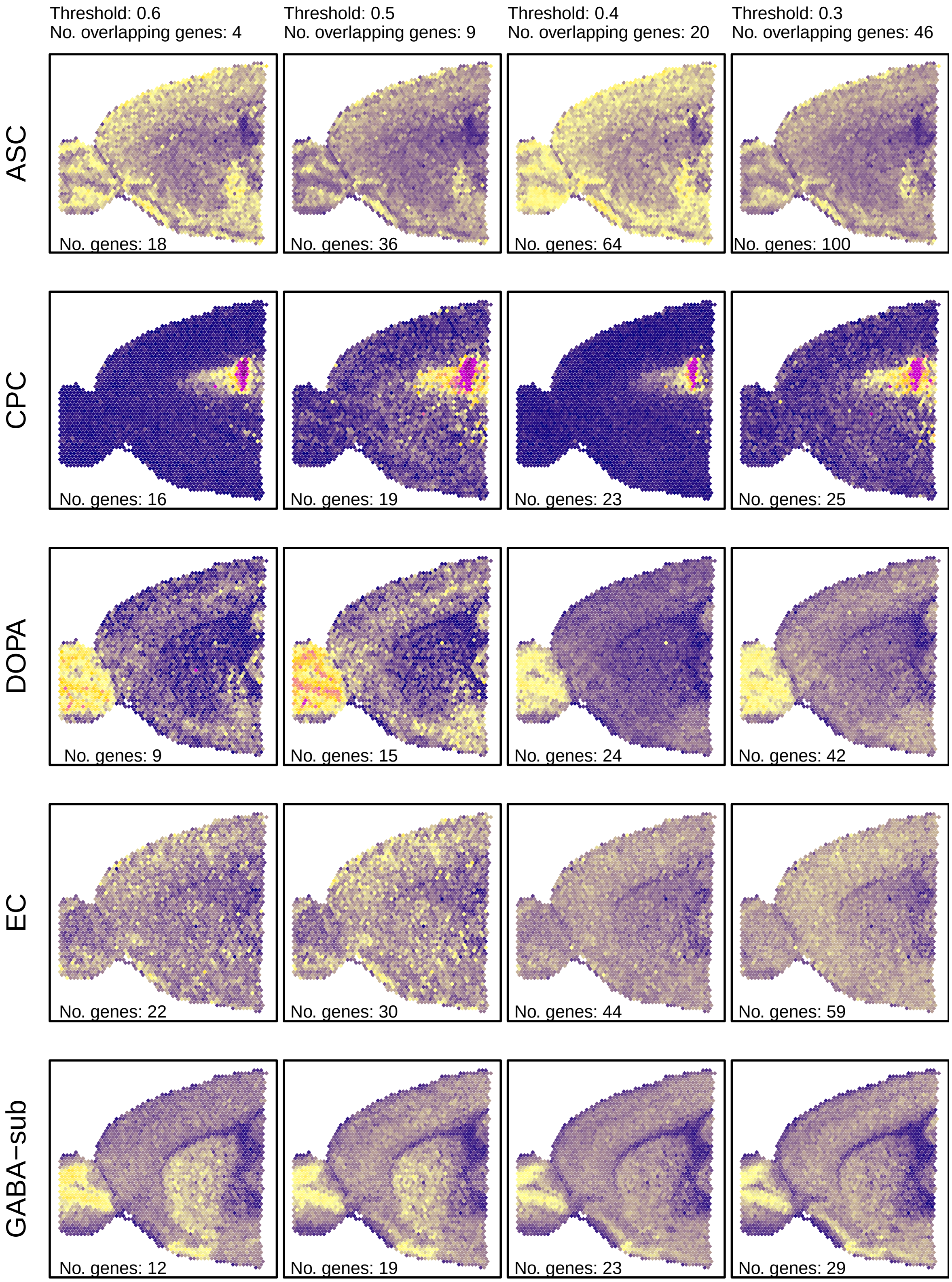

Figure S4: **Results of a study on sensitivity of Celloscope's performance to the choice of marker genes.** Rows indicate different cell types and columns different values of thresholds ($\rho = \tau$, see Methods). At the bottom of each heatmap we denote the number of genes accepted as markers for each cell type.

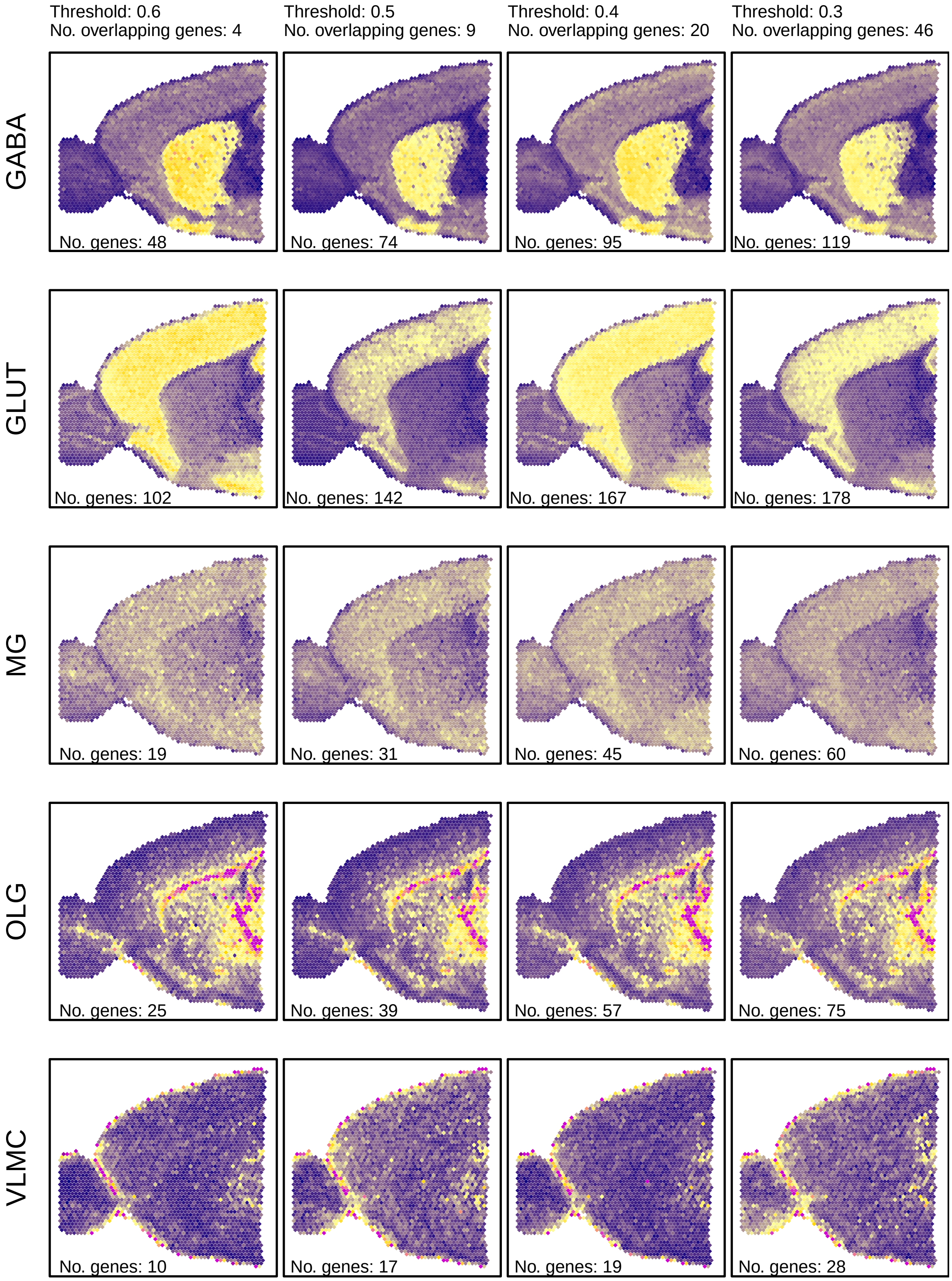

Figure S5: **Results of a study on sensitivity of Celloscope to the choice of marker genes - continued.**

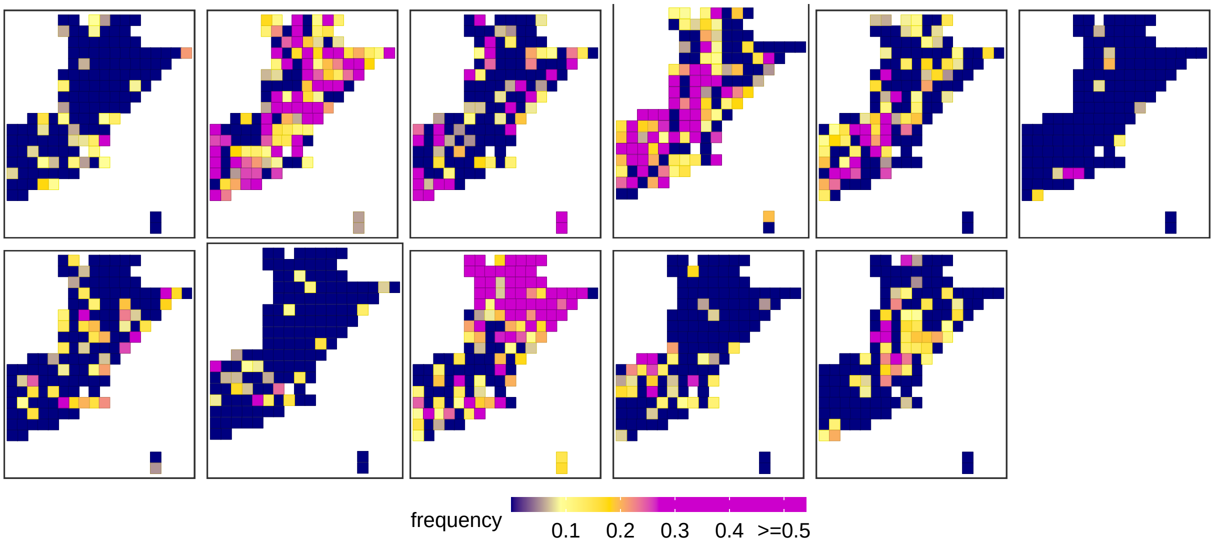

Figure S6: Results obtained with STdeconvolve for human prostate data (section 3.1).

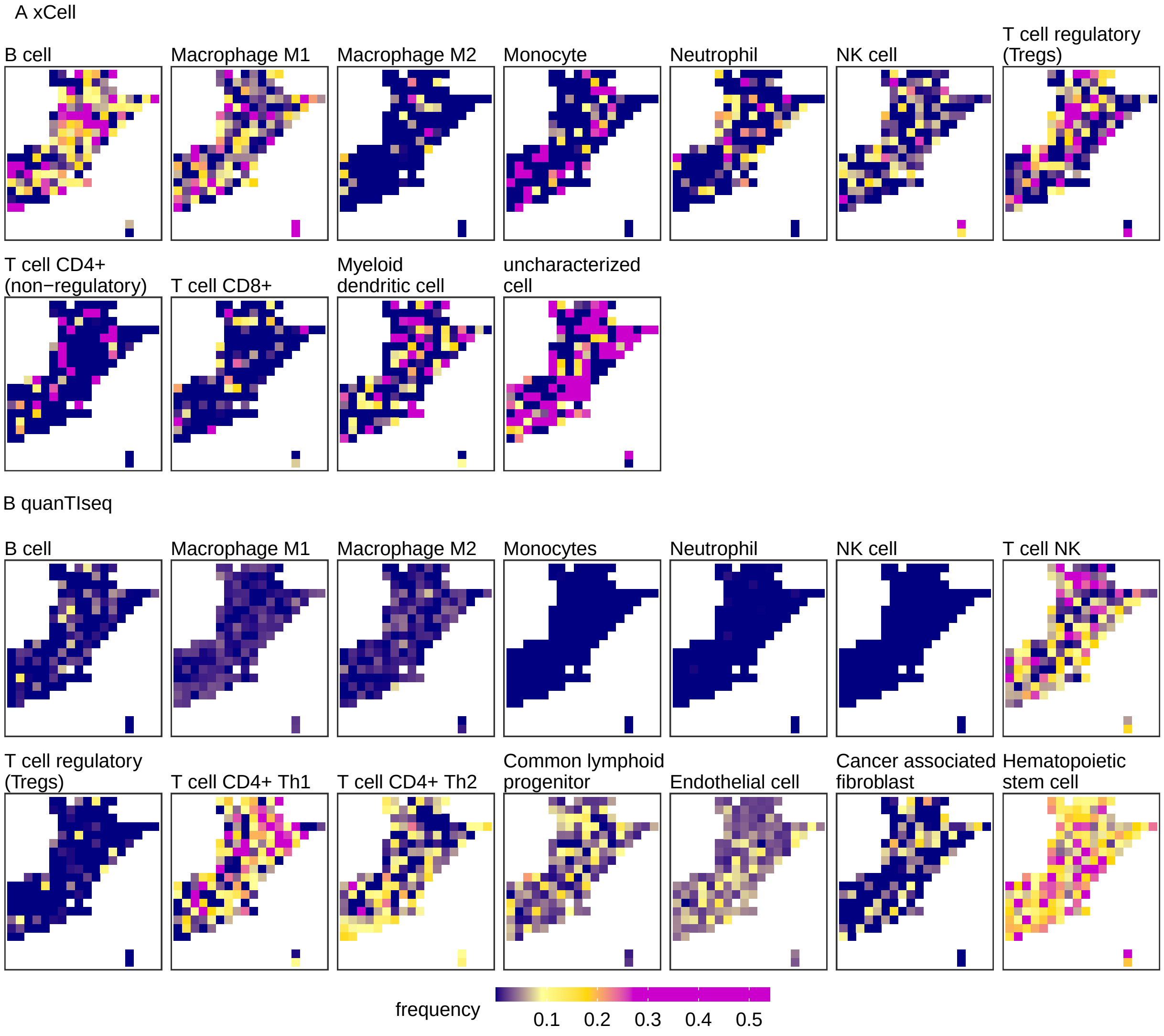

Figure S7: Results of immune cell type decomposition in human prostate data (section 3.1) with the use of (**A**) quanTIseq (the parameter *tumor* was set to *negative*) and (**B** xCell). Results are in a major disagreement. Results obtained wih ABIS and EPIC were not included due to returning a matrix of cell type fractions with 58% negative entries (in the case of ABIS) and due to assigning 66% of the spots on average to uncharacterised cell type in the case of EPIC.

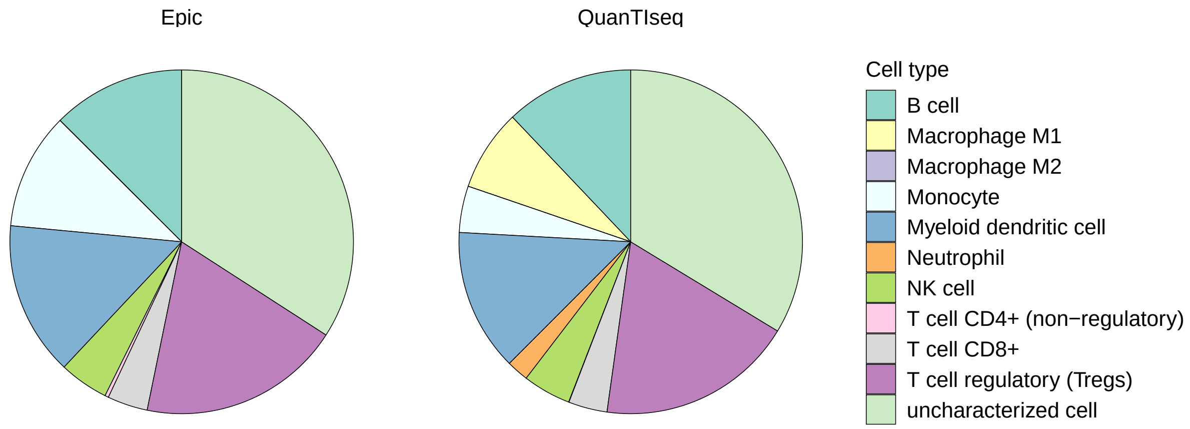

Figure S8: Results of immune cell types decomposition of pooled human prostate data (section 3.1) with the use of EPIC and quanTIseq. For each spot, gene expression in ST data was summed over genes to produce *pseudo bulk*.

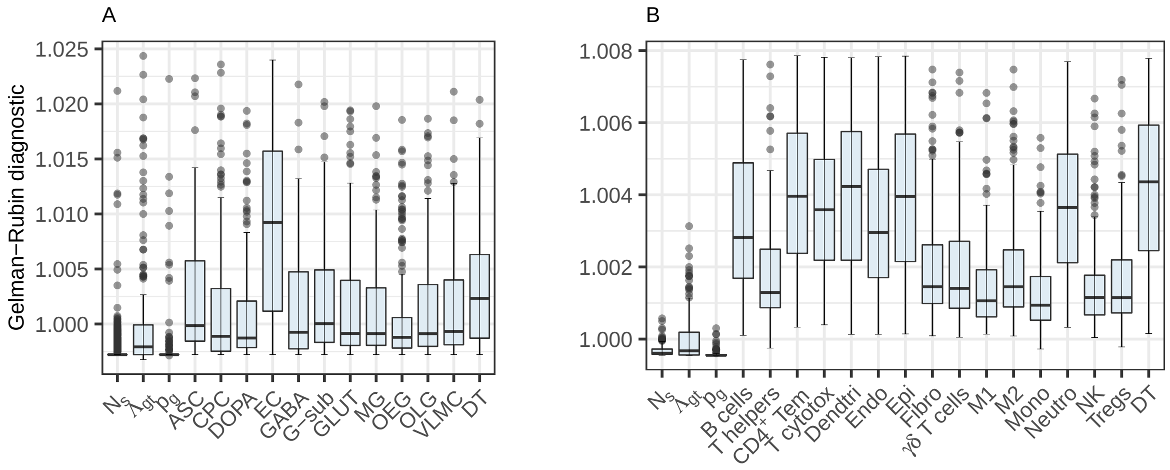

Figure S9: Box-plots represent values for Gelman-Rubin diagnostics test (R package stableGR [64]) with the division into model's variables for mouse brain (**A**) and human prostate data (**B**). In all cases the values are lower than the most commonly used convergence-indicating threshold: 1.1.

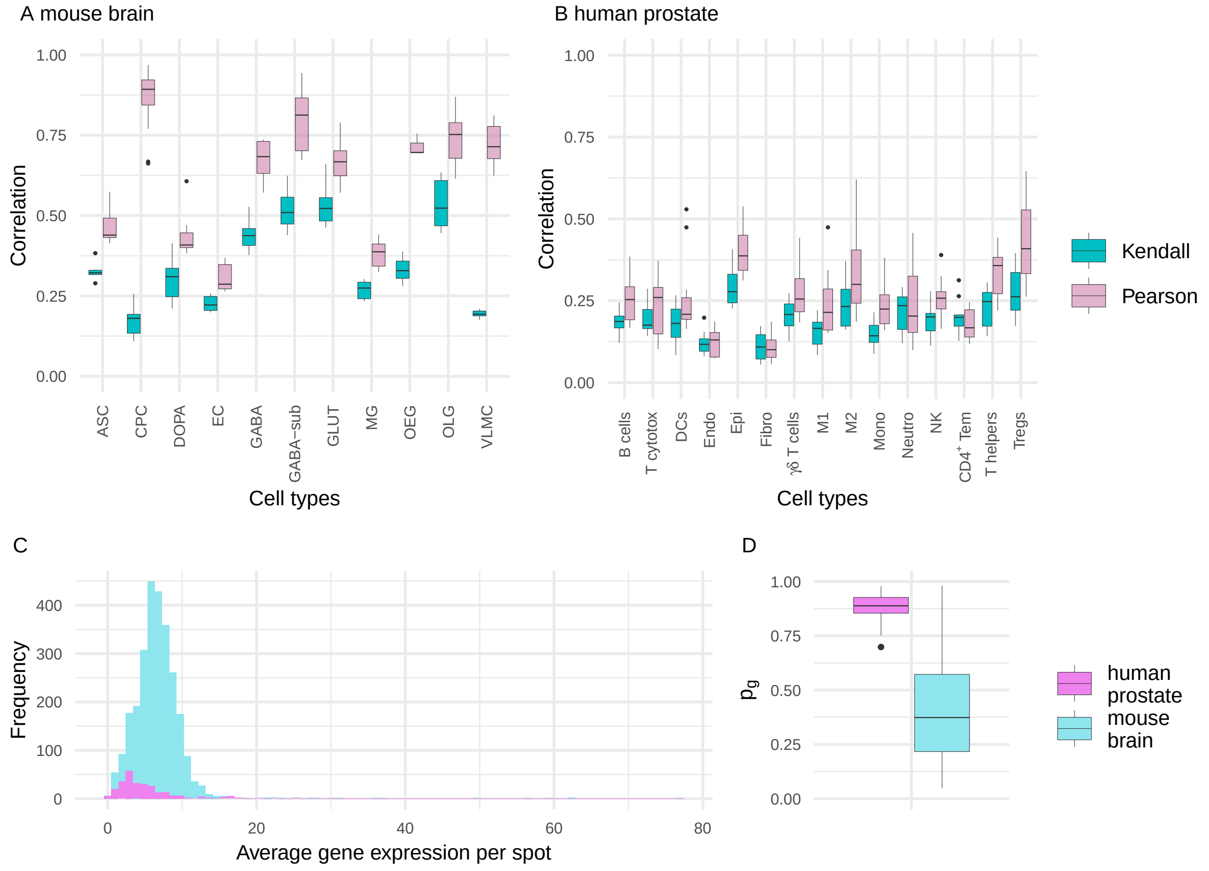

Figure S10: **A, B** Box-plots represent Kendall rank correlation coefficient and Pearson correlation coefficient between expression of the core marker gene (Methods) and the rest genes chosen in the semi-automatic procedure as genes marker for a given cell type. We observe much greater consistency in marker gene expression for mouse brain data (sagittal section) (**A**) than for prostate cancer data (**B**). **C** Histograms represent average gene expression per spot (over genes) in mouse brain data (sagittal section) and human prostate data. We observe much higher coverage for mouse brain data (sagittal section). **D** Boxplots represent estimated values of p_g variable (accounting for over-dispersion) for mouse brain data (blue) and human prostate data (violet). Post-factum analyses reveals much higher over-dispersion in human prostate data than in mouse brain data.

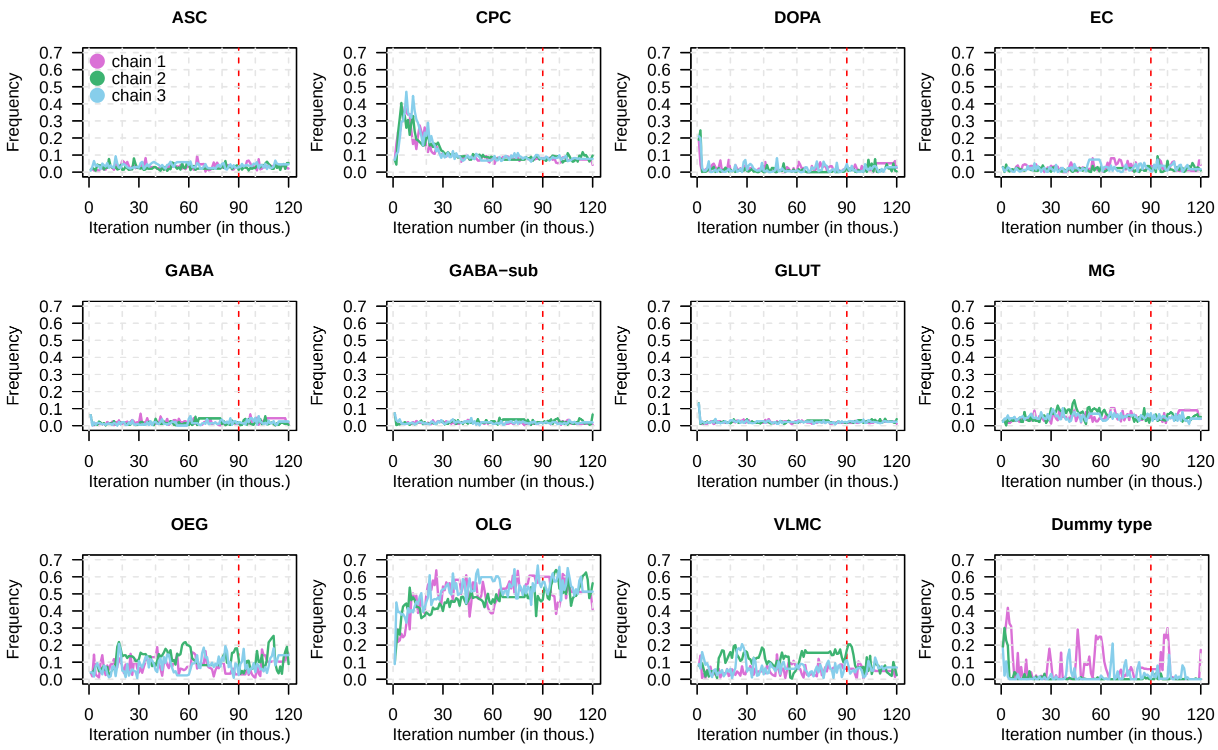

Figure S11: An exemplary trace plot for all cell types proportions for a selected ST spot from mouse brain data (sagittal section). Colors (pink, green, blue) indicate independent chains. Vertical red lines cut off burn-in period.
