# Supplementary material for: Celloscope: a probabilistic model for marker-gene-driven cell type deconvolution in spatial transcriptomics data
Source: Genome Biol. 2023 May 17;24:120. doi: 10.1186/s13059-023-02951-8 (PMC10190053; doi:10.1186/s13059-023-02951-8)
Supplement: Supplementary file 5 — Additional file 5. Review history. [file 13059_2023_2951_MOESM5_ESM.docx]

Review History

**First round of review**

**Reviewer 1**

**Were you able to assess all statistics in the manuscript, including the appropriateness of statistical tests used?**

**Yes:** see main comments

**Were you able to directly test the methods?**

No

**Comments to author:**

Overview

The authors present a new method for spot-level cell type deconvolution in spatial transcriptomics (ST) data, named Celloscope. The innovation is that this method relies on externally sourced lists of marker genes to deconvolve cell types per spot, instead of a reference single-cell RNA sequencing (scRNA-seq) dataset. In principle, this is a useful contribution to the field, which would allow spot-level cell type deconvolution to be applied in datasets where marker gene lists are available but a matched scRNA-seq reference dataset is not available. However, there are several important limitations, which are not fully discussed. First, marker gene lists are only provided for two systems (mouse brain and human prostate cancer), and the procedures for generating these lists are highly customized, so the method cannot easily be applied to other systems. Second, the method assumes the number of cells per spot is provided as an input, but no code is provided for generating this. Third, the two datasets described in the manuscript were analyzed with customized parameter values, and no advice is provided for selecting these parameters in new datasets. Fourth, alternative reference-free methods for spot-level cell type deconvolution are not mentioned. Fifth, no estimates of runtimes and computational complexity are provided.


Major comments

* Marker gene lists are provided for two systems (mouse brain and human prostate cancer), which were generated using customized procedures, which included sourcing gene lists from the literature followed by further curation to select markers with non-zero expression and spatial co-localization in these two datasets. These procedures are not explained in sufficient detail to allow users to apply them in new systems, making it difficult for users to apply the method in new systems. In addition, it is not clear how sensitive the results are to the choice of marker genes.

* The number of cells per spot is assumed to be provided as an input. For the two datasets described (mouse brain and human prostate cancer), these numbers were generated using the QuPath software package with a "custom script extending upon the functionality of QuPath" along with additional manual tuning of parameters (see Methods, section "Cell counting"). This code and parameter values are not provided for users, so it will be difficult for users to apply the method to their own datasets.

* The two datasets (mouse brain and human prostate cancer) were analyzed with manually tuned parameter values, with different values for each dataset (see Methods, sections "Adaptive step size" and "Run setups for mouse brain and human prostate data"). It is not clear how these parameter values would be selected for new datasets to allow users to run the method on their own datasets.

* Alternative reference-free methods for spot-level cell type deconvolution (in particular STdeconvolve, Miller et al. (2022), Nature Communications) are not mentioned.

* Runtimes and computational complexity of the method are not discussed, which is crucial for users to apply the method, especially for an MCMC-based method, which tend to be computationally intensive.


Minor comments

* Background, line 50: mentions that ST spots contain 10-100 cells. Depending on the tissue and platform, this is not accurate. For example, 10x Genomics Visium data in the human brain has been observed at around 3 cells per spot, and the HDST platform referred to in the text in reference [6] has 2um resolution spots, which may be subcellular resolution depending on the tissue.

* Supplementary Note section S2 mentions that the implementation of RCTD used in the benchmarking comparisons was adapted from the original version of RCTD. However, the code for this adapted method is not provided. It is not clear how different this adapted version of RCTD is from the original version.

* Table 1: mentions variable C_gs but the description in the table refers to "cell c"

* Page 2, line 6: typo, "RCTD"

* Page 5, line 24: typo, should this refer to CellAssign?

* Page 5, line 56: typo, "Nissl-staining"

**Reviewer 2**

**Were you able to assess all statistics in the manuscript, including the appropriateness of statistical tests used?**

**Yes:** A detailed review of the probabilistic model would require someone with extensive expertise on the topic as its degree of complexity is high. Based on my expertise and the consolidated experience of this group on the subject, I don't think additional reviews are needed, unless concerns are raised by the other reviewers.

**Were you able to directly test the methods?**

No

**Comments to author:**

The work by Geras and colleague enters a crowded and dynamic field of investigation that is concerned with addressing one of the main challenge of current spatial transcriptomics (ST) technologies: the lack of single-cell resolution. Indeed, ST typically generates spatially resolved expression profiles for tissue "spots", each comprising in the order of tens cells (although this is expected to change in the next 1/2 years). Several deconvolution approaches have hence been developed to resolve cell type composition within each spot.

Here the authors distinguish themselves by developing a rigorous probabilistic model (called Celloscope) to infer cell type proportions without requiring matching single-cell RNA-seq data, as many (not all) other approaches instead do. They compared Celloscope with two others tools on simulated data and tested it on real data from mouse brain and prostate cancer tissues. Overall, the approach is well-designed and of interest and the results are promising. However, while the probabilistic model is described in details, other components of Celloscope (cell segmentation and markers determination) are not sufficiently described and tested. Similarly, comparisons and tests on actual data seem quite limited given the current availability of methods and datasets.

Main comments:

1) For what concerns the method, Celloscope assembles different components: cell counting from H&E slides, cell type markers classifications (pre-determined) and cell type composition inference. The latter is the core of the method and is described in detail in the method section, less so the others. Here I have a few comments:

- Cell segmentation and counting is done with a custom script that the authors developed for QuPath. However, the problem is not straightforward and several approaches have been developed for this purpose (e.g. see StarDist). The authors should: first assess the accuracy of their approach compared to others and in case consider integrating a different cell counting approach into their pipeline, second they should assess how much the performance of their approach depend of cell counting accuracy (it might well be that Celloscope is highly robust to this input, in that case all the better).

- Similarly, the performance of Celloscope should be assessed with respect to the cell type markers used in input. The authors correctly state that this information is largely available in the literature and can be used to denote cell types (as a matter of fact most deconvolution approaches for bulk RNA-seq follow the same principle). However, cell type signatures can vary significantly in size (from 2/3 to dozens of genes) and can or not overlap (as the authors state in the Methods). Do these features affect the method's performances? In particular, the authors say that disjoint cell marker sets are preferable, but to what extent overlapping gene sets affect the performances?
Since cell markers selection is left to the user, this will be important information to provide and, thus, the authors should assess these dependencies in both simulated and real data.

- As a minor note, I am unclear about the definition of the lambda zero parameter (base gene expression) and, even more, about the over-expression parameter (capital lambda). These are initially sampled using the MH-within-Gibbs procedure, but are then set as fixed? I.e. are there two possible expression states for all genes? Couldn't they be fixed a priori? (Maybe-likely I'm missing something here, it would be great if the authors can explain a bit better how these variables are defined and estimated)

2) For what concerns instead the results, I think more can be done to prove the effectiveness and usefulness of the approach.

- First, additional tools could have been considered, e.g. STdeconvolve has been recently published but was in bioRxiv before or SpatialDWLS, which takes uses cell type signatures similarly to what the authors propose. These and the already adopted tools should have been compared also in the context of real data and the results obtained with Celloscope and others compared. Indeed, the comparison with simulated data, while useful, does not provide a realistic use case.

- Similarly, it could be interesting to assess the importance of having matched scRNA-seq data. Indeed, I understand the argument that it might not always be possible but, as a end user, I might be interested in knowing whether doing that extra experiment will significantly improve the results, or if, by using Celloscope, I can save the money and avoid that extra-experiment.
The authors could compare the performance of Celloscope with some recent approaches (e.g. BayesPrism or Cell2location) on datasets where scRNA-seq is also available to quantify the loss in performance (if any) due to not using scRNA-seq information.

- Finally the testing on real datasets is quite limited and it would be nice to see more extensive analyses. For the mouse brain experiment, several visium slides are available, it would be important to show the consistency of performance across this dataset. Similarly for the prostate cancer dataset, the authors say 12 slides are available, why selecting only one and a portion of it? Again consistency of spatial correlations and/or recurrent cell type compositions could be explored.

- In particular, the prostate cancer example might benefit from additional analysis on additional slides as the results currently presented are hard to grasp. For example, the authors comment on the enrichment of endothelial and fibroblast cells in specific areas, but I cannot honestly see what they are referring to. The colors on Fig. 4A seem rather uniform across the region with minor differences and panel B is too small and not annotated to allow the reader to appreciate these enrichments. Moreover, additional quantitative analyses should be done to quantify and prove a spatial enrichment. Similarly, it is unclear what supports the conclusions made in the whole last paragraph of this section (top of page 10 "The composition of cell types identified using Celloscope.."). Is this based on prior knowledge? What are the images corresponding to what the authors are describing?

- While it could require work beyond the scope of this study, have the authors considered the possibility that inference of cell type composition in one spot could be informed by inferred cell type compositions of the other spots? Specific compositions might be "recurrent" and hence one might favor solutions that limit the number of diverse unique compositions across multiple spots? (Having multiple slides/samples for the same tissue/condition could here be exploited). But again this might go beyond the scope, I just wonder whether this has been considered and what are the authors' thoughts on this regard.

Minor:

3) Figures need improvement especially Figure 3 and 4. In the panels A of both figures, colored dots often overlap, the effect is particularly evident in Figure 4A. This should be avoided as it is difficult to appreciate the spatial composition and since spots are disjoint a better representation can be used (e.g. previous Visium slide representations used a hexagonal mesh to mimic the spots distribution)

4) Figures are also erroneously referenced: e.g. Fig. 3D and 3E are cited before Fig 3C and do not really describe the corresponding figure panels, a similar situation is for Figure 4. Double-check all figure references

5) I do not understand the "X" notation in the correlation plots in Fig. 3E and 4F: the authors say it indicates non-significant correlations, does that imply that all the other correlation values are significant? that seems unlikely since there are white or very light spots in the heatmap with correlation values close to zero..

6) Some phrasing might be simpler, e.g. "most dominant" seems redundant given "dominant" already indicate the cell type present with the highest proportion, "immense infiltration" sounds excessive..etc.

**Reviewer 3**

**Were you able to assess all statistics in the manuscript, including the appropriateness of statistical tests used?**

**Yes:** No concerns about the statistics.

**Were you able to directly test the methods?**

Yes

**Comments to author:**

Geras et al. developed Celloscope, a Bayesian probabilistic graphical model, which deconvolutes cell type composition in spatial transcriptomics data. The authors have demonstrated the main advantage of Celloscope: it does not rely on single-cell NA-seq data as reference due to the use of quantitative information of cell type specific marker genes. They showed that the proposed methods outperformed others in four different simulation scenarios. In addition, Celloscope was able to spatially distinguish between two subclasses of neurons in a real mouse brain ST data, and to delineates the spatial coordinates immune infiltration in human prostate tissues. Altogether, the results of this study are impressive. Still, some clarifications are needed and a few further questions should be addressed:

First, the proposes method heavily depends on the choice of cell type specific markers. The manuscript provided the marker lists only for mouse brain and human prostate tissue, based on the spatial agreement of their expression across ST spots. However, there are other factors that impact the accuracy of ST deconvolution. One is the cell plasticity, which results in a continuum of immune phenotypes coexistence within the same system, complicating the distinction between "cell type" and "cell state". Another factor that many methods neglected is that immune cells can exhibit tumor/tissue-specific aberrant expression. It will be great if the authors can provide more details on marker selection and discuss how to address these issues with proposed method.

Second, a dummy type was incorporate in the mode to account for cell type that exists in spot but is not represented by the cell type specific marker. However, the author didn't evaluate the algorithm's performance in scenarios when unknown cell types exist in ST spots.

Third, since the proposed method is marker-dependent without using any matched single-cell RNA-seq data, how is it different from other marker-dependent deconvolution methods developed for bulk transcriptomics data (non-spatial)? It will be interesting to see a comparison between Celloscope and methods for bulk transcriptomics data deconvolution, such as TIMER, EPIC, and quanTIseq.

Finally, the proposed methods, or any method benchmarked in this study, make explicit use of the spatial coordinates during modeling. The authors should at least discuss how the inference of cell types in a given spot should be influenced by the nearby spot.

**Authors Response**

We are grateful to the Reviewers for their comments. We have modified the manuscript accordingly. This revision resulted in a significant **extension of one Figure**, **addition of 2 new Figures** and **13 new subsections** in the main text; we also added **10 new Supplementary Figures**, **3 new sections** to Additional File 1 and **one new** Additional File. We believe that this has led to a large improvement of the manuscript.

**Point-by-point responses to the reviewers’ comments:**

*Reviewer #1: Overview*

*The authors present a new method for spot-level cell type deconvolution in spatial transcriptomics (ST) data, named Celloscope. The innovation is that this method relies on externally sourced lists of marker genes to deconvolve cell types per spot, instead of a reference single-cell RNA sequencing (scRNA-seq) dataset. In principle, this is a useful contribution to the field, which would allow spot-level cell type deconvolution to be applied in datasets where marker gene lists are available but a matched scRNA-seq reference dataset is not available. However, there are several important limitations, which are not fully discussed. First, marker gene lists are only provided for two systems (mouse brain and human prostate cancer), and the procedures for generating these lists are highly customized, so the method cannot easily be applied to other systems. Second, the method assumes the number of cells per spot is provided as an input, but no code is provided for generating this.*

*Third, the two datasets described in the manuscript were analyzed with customized parameter values, and no advice is provided for selecting these parameters in new datasets. Fourth, alternative reference-free methods for spot-level cell type deconvolution are not mentioned. Fifth, no estimates of runtimes and computational complexity are provided.*

*Major comments*

***Comment 1:*** *Marker gene lists are provided for two systems (mouse brain and human prostate cancer), which were generated using customized procedures, which included sourcing gene lists from the literature followed by further curation to select markers with non-zero expression and spatial co-localization in these two datasets. These procedures are not explained in sufficient detail to allow users to apply them in new systems, making it difficult for users to apply the method in new systems. In addition, it is not clear how sensitive the results are to the choice of marker genes.*

**Response:** Thank you for this comment. To address this issue, we devised, implemented and described in detail a semi-automatic procedure for marker gene selection. This procedure filters out genes with low expression and selects marker genes based on their co-expression in spots, as quantified by the Kendall and Pearson correlation coefficients. The procedure is described in a new subsection **“Defining the marker genes for cell types”** in the main text, its source code in R is available in the GitHub repository and it was used to generate results in the manuscript **(Figures 3, 4, 5, 6)**.

To test Celloscope’s sensitivity to marker gene choice in the context of real data, we run Celloscope for sagittal mouse brain data with the use of different sets of marker genes,

acquired with different parameters of the semi-automatic procedure for marker gene selection. These experiments confirmed that Celloscope is robust to the choice of the marker genes. Results are available in the manuscript in a new subsection “**Sensitivity to the choice of marker genes”** in the main text and **Additional File 1: Figure S4 and S5**. As the thresholds of the procedure decrease, the procedure becomes less restrictive, more genes are accepted, and we observe a higher number of overlapping genes in marker gene sets. Therefore, in this experiment we not only investigate the sensitivity to changes in parameters of the semi-automatic procedure, but also we investigate if overlapping gene sets affect the performance in the context of real data.

Additionally, we performed a study on simulated data to test whether overlapping marker gene sets affect Celloscope’s performance, again showing that Celloscope is robust to marker gene overlaps. Results are featured in a new **Figure 2C** and are discussed in the subsection **“Overlapping marker gene sets do not affect Celloscope's performance on simulated data”.**

***Comment 2:*** *The number of cells per spot is assumed to be provided as an input. For the two datasets described (mouse brain and human prostate cancer), these numbers were generated using the QuPath software package with a "custom script extending upon the functionality of QuPath" along with additional manual tuning of parameters (see Methods, section "Cell counting"). This code and parameter values are not provided for users, so it will be difficult for users to apply the method to their own datasets.*

**Response:** The custom script for counting the number of cells per spot is now available in our **GitHub repository (**[**https://github.com/szczurek-lab/qupath-spot-utils)**](https://github.com/szczurek-lab/qupath-spot-utils)) with clear instructions on how to run it after installing QuPath. Default values of parameters and provided in the script. Additionally, some advice on parameter tuning is given in the manuscript in the subsection **“Recommendation on parameters choice on new data sets: Cell counting”** along with reference to the QuPath’s tutorial, where detailed instructions on how to choose parameters are given.

***Comment 3:*** *The two datasets (mouse brain and human prostate cancer) were analyzed with manually tuned parameter values, with different values for each dataset (see Methods, sections "Adaptive step size" and "Run setups for mouse brain and human prostate data"). It is not clear how these parameter values would be selected for new datasets to allow users to run the method on their own datasets.*

**Response:** Thank you for this remark. We added a new subsection **“Recommendation on parameters choice on new data sets: Running Celloscope”**, where we instruct how the parameters should be selected.

***Comment 4:*** *Alternative reference-free methods for spot-level cell type deconvolution (in particular STdeconvolve, Miller et al. (2022), Nature Communications) are not mentioned.*

**Response:** Thank you for this suggestion. STdeconvolve is now not only mentioned and cited, but also was applied on human prostate and mouse brain data and its results are compared to the ones obtained using Celloscope. The outcomes of running STdeconvolve on the same data are featured in three new Figures, **(Additional file 1: Figures S1, S3, S6)**

and are discussed and compared to Celloscope in three new subsections **“Comparison to STdeconvolve for the sagittal mouse brain section”**, **“Comparison to STdeconcolve and SpatialDWLS for the coronal mouse brain section”** and **“Comparison to STdeconvolve for human prostate data"**in the main text. It is important to note that detailed, quantitative comparison of STdeconvolve and Celloscope is difficult due to the fact that, due to its unsupervised nature, as a topic model, STdeconvolve outputs uncharacterized and un-annotated cell types in the form of the identified ‘topics’. The task of mapping the topics detected by STdeconcolve to cell types assumed by Celloscope was only possible to some extent, by visual inspection and matching the annotated cell types found by Celloscope to those expressed in similar areas and found using STdeconvolve. Our analysis indicated that thanks to prior knowledge of different cell types and their markers, Celloscope can provide a more fine-grained deconvolution into cell types than STdeconvolve.

***Comment 5:*** *Runtimes and computational complexity of the method are not discussed, which is crucial for users to apply the method, especially for an MCMC-based method, which tend to be computationally intensive.*

**Response:** Thank you for this remark. We now included a new subsection **“Run-times and computational complexity of Celloscope”** in the main text**,** where we discussed those two matters.

*Minor comments*

***Comment 6:*** *Background, line 50: mentions that ST spots contain 10-100 cells. Depending on the tissue and platform, this is not accurate. For example, 10x Genomics Visium data in the human brain has been observed at around 3 cells per spot, and the HDST platform referred to in the text in reference [6] has 2um resolution spots, which may be subcellular resolution depending on the tissue.*

**Response:** Thank you for pointing this out. This fragment from the Introduction was modified and the inaccuracy corrected according to your remark. Now it states that: “***ST spots differ in resolution depending on the tissue and technology from subcellular, through 1-10, up to 100 cells per spot”****.* It is also important to mention here that our own cell counting procedure, which was very carefully curated and validated by manual counting of cellular nuclei, revealed the mean number of cells of the order of 10 cells per spot for the mouse brain data and 77 per spot for the prostate data. These values underline the challenge posed by at least some of the current spatial transcriptomics (ST) technologies: the lack of single-cell resolution, and the need for the deconvolution of spots into cell types.

***Comment 7:*** *Supplementary Note section S2 mentions that the implementation of RCTD used in the benchmarking comparisons was adapted from the original version of RCTD. However, the code for this adapted method is not provided. It is not clear how different this adapted version of RCTD is from the original version.*

**Response:** Thank you for noticing this inconsistency. That was an overstatement, which is now corrected in the revised manuscript. RCTD did not need to be adapted. The code and

commands were already available. All run settings for methods used previously (Additional File 1 Sections S1, S2, S4) and added during the revision (Additional File 1 Sections S3, S5, S6) are provided.

***Comment 8:*** *Table 1: mentions variable C_gs but the description in the table refers to "cell* *c"*

**Response:** Thank you, the typo was corrected to “spot s”.

***Comment 9:*** *Page 2, line 6: typo, "RCTD"*

**Response:** Thank you, the typo was corrected.

***Comment 10:*** *Page 5, line 24: typo, should this refer to CellAssign?*

**Response:** Actually, this indeed refers to CellAssign. **Figure 2B** presents accuracy in which methods designed for cell type deconvolution were able to indicate the dominant cell type (for each spot, the cell type with the largest proportion according to the true, simulated values of variable H and the inferred values of *H*). These accuracies are compared to the accuracy of CellAssign, which aimed to assign a single type to each spot, without accounting for the mixture of cells in each spot. The accuracy for CellAssign was computed as the fraction of times when CellAssign assigned the spots to a cell type that was the same as the cell type with the largest proportion according to the true, simulated values of variables *H*. As it turned out, both Celloscope and the competitive deconvolution methods indicated the dominant type with higher accuracy than CellAssign. The description of this analysis was now turned into a separate subsubsection entitled **“Accounting for cell-type mixtures in spots increases model’s accuracy”** and extended.

***Comment 11:*** *Page 5, line 56: typo, "Nissl-staining"*
**Response:** Thank you, the typo was corrected.

*Reviewer #2:*

*The work by Geras and colleague enters a crowded and dynamic field of investigation that is concerned with addressing one of the main challenge of current spatial transcriptomics (ST) technologies: the lack of single-cell resolution. Indeed, ST typically generates spatially resolved expression profiles for tissue "spots", each comprising in the order of tens cells (although this is expected to change in the next 1/2 years). Several deconvolution approaches have hence been developed to resolve cell type composition within each spot.*

*Here the authors distinguish themselves by developing a rigorous probabilistic model (called Celloscope) to infer cell type proportions without requiring matching single-cell RNA-seq data, as many (not all) other approaches instead do. They compared Celloscope with two others tools on simulated data and tested it on real data from mouse brain and prostate cancer tissues. Overall, the approach is well-designed and of interest and the results are promising. However, while the probabilistic model is described in details, other components of Celloscope (cell segmentation and markers determination) are not sufficiently described and tested. Similarly, comparisons and tests on actual data seem quite limited given the current availability of methods and datasets.*

*Main comments:*

*1) For what concerns the method, Celloscope assembles different components: cell counting from H&E slides, cell type markers classifications (pre-determined) and cell type composition inference. The latter is the core of the method and is described in detail in the method section, less so the others. Here I have a few comments:*

***Comment 1:*** *Cell segmentation and counting is done with a custom script that the authors developed for QuPath. However, the problem is not straightforward and several approaches have been developed for this purpose (e.g. see StarDist). The authors should: first assess the accuracy of their approach compared to others and in case consider integrating a different cell counting approach into their pipeline, second they should assess how much the performance of their approach depend of cell counting accuracy (it might well be that Celloscope is highly robust to this input, in that case all the better).*

**Response:** Thank you for this suggestion. We performed a comprehensive study on simulated data to test Celloscope’s robustness to noise in cell counts estimation results. The results are featured in an extended **Figure 2A** and are discussed in a new subsection **“Celloscope is robust to noise in cell counts estimation results”**.

To our best knowledge, StarDist is indeed designed to analyze H&E images, and is ready to count all cells detected on an input H&E image, but not inside the ST spots, which have specific locations and radius. Thus, StarDist does not provide the functionality that is necessary to count cells inside ST spots, which does not allow us to compare our cell counting procedure to this method.

Our own evaluation included careful comparison of the outcomes of our script with manual cell counting in randomly chosen spots by eye. Also, it is important to know that the accuracy of cell counting depends on parameter settings for the cell detection algorithm inside QuPath. To make it easier for the users to adjust these parameters for their specific H&E images, which may have different contrast, noise or color scale, we provided a new subsection **“Recommendation on parameters choice on new data sets: Cell counting”,** along with reference to the QuPath’s tutorial, where detailed instructions on how to choose parameters are given.

***Comment 2:*** *Similarly, the performance of Celloscope should be assessed with respect to the cell type markers used in input. The authors correctly state that this information is largely available in the literature and can be used to denote cell types (as a matter of fact most deconvolution approaches for bulk RNA-seq follow the same principle). However, cell type signatures can vary significantly in size (from 2/3 to dozens of genes) and can or not overlap (as the authors state in the Methods). Do these features affect the method's performances? In particular, the authors say that disjoint cell marker sets are preferable, but to what extent overlapping gene sets affect the performances?*

*Since cell markers selection is left to the user, this will be important information to provide and, thus, the authors should assess these dependencies in both simulated and real data.*

**Response:** To test the Celloscope’s sensitivity to marker gene choice, we first performed a study on simulated data to assess the influence of overlapping marker gene sets. The results are featured in a new **Figure 2C** and discussed in the subsection **“Overlapping**

**marker gene sets do not affect Celloscope’s performance on simulated data”** in the main text**.**

To make the process of defining marker genes for each cell type easier and more automated for the user, we devised, implemented and described in detail a semi-automatic procedure for marker gene selection. This procedure filters out genes with low expression and selects marker genes based on their co-expression in spots, as quantified by the Kendall and Pearson correlation coefficients. The procedure is described in a new subsection **“Defining the marker genes for cell types”** in the main text, its source code in R is available in the GitHub repository and it was used to generate results in the manuscript **(Figures 3, 4, 5, 6)**.

Finally, we investigated Celloscope’s sensitivity to marker gene choice with the use of mouse brain sagittal section data. To this end, we run Celloscope with different sets of marker genes, acquired using different parameters of the semi-automatic procedure for marker gene selection (Methods). We now discuss that Celloscope is robust to the differences in marker gene selection in a new subsection “**Sensitivity to the choice of marker genes”,** visualizing the results in **Additional File 1: Figure S4 and S5**. As the thresholds of the procedure decrease, the procedure becomes less restrictive, more genes are accepted, and we observe a higher number of overlapping genes in marker gene sets. Therefore, in this experiment we do not only investigate the sensitivity to changes in parameters of the semi-automatic procedure, but also we investigate if overlapping gene sets affect the performance in the context of real data.

***Comment 3:*** *As a minor note, I am unclear about the definition of the lambda zero parameter (base gene expression) and, even more, about the over-expression parameter (capital lambda). These are initially sampled using the MH-within-Gibbs procedure, but are then set as fixed? I.e. are there two possible expression states for all genes? Couldn't they be fixed a priori? (Maybe-likely I'm missing something here, it would be great if the authors can explain a bit better how these variables are defined and estimated).*

**Response:** Thank you for stressing this problem. We added an additional passage clarifying lambda zero parameter definition in **Methods (page 22)**.

You are right, there are two possible states of expression, either gene is a non-marker and its expression is lambda zero (base expression); or gene is a marker and we add to lambda zero an additional term accounting for over-expression (capital lambda, that is dependent on the gene in question). These states are indeed fixed a priori with the use of matrix B that indicates if genes are markers for the expected cell types.

In contrast, the values of lambda zero and of the capital lambda are by default not fixed in Celloscope, but are hidden variables and inferred by the model. For the formal explanation of the variable inference see **Algorithm 1** in the manuscript, line 6 and 7, clarifying how capital lambda and lambda zero are updated in every iteration.

Fixing capital lambda and lambda zero could be possible by clamping them to observed values when they would be available. We now experimented with fixing them in the new simulation settings (**Figure 2A**), showing that fixing them to true values increases model performance, but only a little bit (subsection “**Knowledge of gene expression profiles of**

**cell types does not have a large impact on model accuracy**”), since the model is able to infer them reliably.

The fact that Celloscope is able to infer the values of lambda zero and capital lambda is key for dealing with the lack of external data sets such as scRNA-seq data, based on which these values could be estimated. Some alternative approaches fix the variables corresponding to gene expression in their models to an average of expression of the genes across the spots; such an approach is wrong given the fact that each spot can be a mixture of different cell types.

*2) For what concerns instead the results, I think more can be done to prove the effectiveness and usefulness of the approach.*

***Comment 4:*** *First, additional tools could have been considered, e.g. STdeconvolve has been recently published but was in bioRxiv before or SpatialDWLS, which takes uses cell type signatures similarly to what the authors propose. These and the already adopted tools should have been compared also in the context of real data and the results obtained with Celloscope and others compared. Indeed, the comparison with simulated data, while useful, does not provide a realistic use case.*

**Response:** Thank you for this suggestion. We now apply STdeconvolve to: mouse brain data sagittal section **(Additional File 1: Figure S1)**, mouse brain data coronal section **(Additional File 1: Figure S3)** and human prostate data, section 3.1 **(Additional File 1: Figure S6)**. We compare those findings to Celloscope’s results in new subsections **“Comparison to STdeconvolve for the sagittal mouse brain section”**, **“Comparison to STdeconcolve and SpatialDWLS for the coronal mouse brain section”** and **“Comparison to STdeconvolve for human prostate data”**.

Apart from that, we made an attempt to compare Celloscope’s performance to STdeconvolve’s outcomes on simulated data. Unfortunately, as an unsupervised topic model, STdeconvolve finds unannotated ‘topics’ that are not assigned to any specific cell type. The task of mapping topics detected by STdeconcolve to the true, simulated cell types turned out to be extremely cumbersome, as some of the topics did not match the simulated ones at all, or there were several that matched a single simulated cell type equally well; resolving this mapping was unfeasible to perform and made the comparison on simulated data impossible.

Moreover, in this revision we included an additional analysis of the mouse brain coronal section data **(Figure 4).** As this data set was previously analyzed by SpatialDWLS in their study (Dong et al, 2021), we were able to additionally compare SpatialDWLS results with the Celloscope’s output. In contrast to Celloscope, SpatialDWLS relies on an external reference dataset in the form of scRNA-seq data. The discussion of these results is available in the main text, in the new subsection **“Comparison to STdeconcolve and SpatialDWLS for the coronal mouse brain section”.** On top of that, we showed increased performance of Celloscope compared to SpatialDWLS on simulated data **(Figure 2A)**.

Finally, we also performed a comparison to BayesPrism on simulated data **(Figure 2D)**. This analysis revealed very low performance of BayesPrism on small sets of marker genes, which

were used for all our other simulations and comparisons with other methods **(Figure 2A, B, C)**. To meet the requirements of the number of marker genes defined by the authors of BayesPrism in the method’s tutorial (“Ideally, we would like to have sufficient number of genes selected for each cell type (>50)”) we prepared a new simulated data set. This analysis indicated that even for larger marker gene sets, Celloscope still outperformed BayesPrism in cell type deconvolution **(Figure 2D)**.

***Comment 5:*** *Similarly, it could be interesting to assess the importance of having matched scRNA-seq data. Indeed, I understand the argument that it might not always be possible but, as a end user, I might be interested in knowing whether doing that extra experiment will significantly improve the results, or if, by using Celloscope, I can save the money and avoid that extra-experiment.*

*The authors could compare the performance of Celloscope with some recent approaches (e.g. BayesPrism or Cell2location) on datasets where scRNA-seq is also available to quantify the loss in performance (if any) due to not using scRNA-seq information.*

To address this question, we first performed a study on simulated data to see if additional information about gene expression levels in different cell types that can be retrieved from scRNA-seq would improve Celloscope’s performance. As visualized in extended **Figure 2A, 2B and 2D,** it turned out that having the gene expression levels improves the model’s performance only a little bit, as it is able to reliably infer gene expression levels when they are not given. Also, this experiment did not take into account bias and technical errors of the potential scRNA-seq reference data, which would negatively influence the performance of the models that would rely on it. The results of this investigation are now discussed in a new subsection **“Knowledge of gene expression profiles of cell types does not have a large impact on model accuracy”**.

On top of that, we performed a comparison to SpatialDWLS, which uses scRNA-seq to compute cell type expression profiles by running this approach for mouse brain coronal section data. As described in a new section ”**Comparison to STdeconcolve and SpatialDWLS for the coronal mouse brain section”** in the main text, the comparison indicated that even without the handicap of reference scRNA-seq data, Celloscope was able to find similar cell types as SpatialDWLS, omitting only those which we did not search for (i.e., did not include in the prior matrix of cell types and their markers).

***Comment 6:*** *Finally the testing on real datasets is quite limited and it would be nice to see more extensive analyses. For the mouse brain experiment, several visium slides are available, it would be important to show the consistency of performance across this dataset. Similarly for the prostate cancer dataset, the authors say 12 slides are available, why selecting only one and a portion of it? Again consistency of spatial correlations and/or recurrent cell type compositions could be explored.*

**Response:** We prepared more extensive analyses and also considered mouse brain data coronal section **(Figure 4 in the main text)**, showing that the results were consistent between these two **orthogonal** sections. We also added results for one more section from human prostate data. Although indeed there were in total 12 slides for the human prostate tissue; only in two of those a non negligible number of spots with immune cells were also indicated by an expert pathologist in their corresponding H&E images **(Figure 6).** Spatial

correlations and recurrent cell types were again explored. Similarly to the mouse brain datasets, the results between the two human prostate sections were in a large agreement, further validating our approach.

***Comment 7:*** *In particular, the prostate cancer example might benefit from additional analysis on additional slides as the results currently presented are hard to grasp. For example, the authors comment on the enrichment of endothelial and fibroblast cells in specific areas, but I cannot honestly see what they are referring to. The colors on Fig. 4A seem rather uniform across the region with minor differences and panel B is too small and not annotated to allow the reader to appreciate these enrichments. Moreover, additional quantitative analyses should be done to quantify and prove a spatial enrichment. Similarly, it is unclear what supports the conclusions made in the whole last paragraph of this section (top of page 10 "The composition of cell types identified using Celloscope.."). Is this based on prior knowledge? What are the images corresponding to what the authors are describing?*

**Response:** The abovementioned conclusions were made on the basis of the detailed visual inspection of the histopathological presentation of the probed prostate specimen, done by the expert clinical pathologist who is a co-author of this paper (Dr Lukasz Koperski). The probed area is localized nearby the urethra (urethral epithelial cells are present here). Squeezed by the hypertrophic prostate urethra is the common source of the prostate infecting pathogens. Moreover, this specimen lacked tumor cells and was composed mostly of the normal epithelium and mononuclear and polynuclear immune cells. The latter can be morphologically identified as neutrophils. The presence of neutrophils in the tissue strongly suggests bacterial (or way less frequently observed – fungal) infection. The mononuclear cells were identified by Celloscope as NK cells. As NK cells are involved mainly in the elimination of intracellular pathogens – the hypothesis of mixed intra- and extracellular infection was made.

In this revision, we improved the visualization in **Figure 5** to help the reader appreciate these enrichments, and we extended the explanation in the text. The additional analyses of section 4.2. is in agreement with the analyses performed previously (section **Celloscope elucidated the source of inflammation in a human prostate tissue** in the manuscript).

***Comment 8:*** *While it could require work beyond the scope of this study, have the authors considered the possibility that inference of cell type composition in one spot could be informed by inferred cell type compositions of the other spots? Specific compositions might be "recurrent" and hence one might favor solutions that limit the number of diverse unique compositions across multiple spots? (Having multiple slides/samples for the same tissue/condition could here be exploited). But again this might go beyond the scope, I just wonder whether this has been considered and what are the authors' thoughts on this regard.*

**Response:** Thank you for giving us this interesting insight. In the **Discussion** of the revised manuscript, we now mention it as a possible extension of the model and future work, referring to two other studies, where information from neighboring spots was taken into consideration to solve tasks in analysis of spatial transcriptomics data but different than cell type deconvolution.

*Minor:*

***Comment 9:*** *Figures need improvement especially Figure 3 and 4. In the panels A of both figures, colored dots often overlap, the effect is particularly evident in Figure 4A. This should be avoided as it is difficult to appreciate the spatial composition and since spots are disjoint a better representation can be used (e.g. previous Visium slide representations used a hexagonal mesh to mimic the spots distribution)*

**Response:** Thank you for pointing out this problem. We improved both figures and the problem of overlapping dots is now avoided.

***Comment 10:*** *Figures are also erroneously referenced: e.g. Fig. 3D and 3E are cited before Fig 3C and do not really describe the corresponding figure panels, a similar situation is for Figure 4. Double-check all figure references.*

**Response:** Thank you for spotting this mistake. It was corrected. We double checked all figure references.

***Comment 11:*** *I do not understand the "X" notation in the correlation plots in Fig. 3E and 4F: the authors say it indicates non-significant correlations, does that imply that all the other correlation values are significant? that seems unlikely since there are white or very light spots in the heatmap with correlation values close to zero.*

**Response:** The X denotes the fact that in the test with the test statistics based on the Pearson’s product moment correlation coefficient, the p-value for the correlation was smaller or equal 0.05.

***Comment 12:*** *Some phrasing might be simpler, e.g. "most dominant" seems redundant given "dominant" already indicate the cell type present with the highest proportion, "immense infiltration" sounds excessive..etc.*

**Response:** Thank you for this remark. The phrase *"most dominant"* was simplified to “dominant” and "immense infiltration" to infiltration.

*Reviewer #3:*

*Geras et al. developed Celloscope, a Bayesian probabilistic graphical model, which deconvolutes cell type composition in spatial transcriptomics data. The authors have demonstrated the main advantage of Celloscope: it does not rely on single-cell NA-seq data as reference due to the use of quantitative information of cell type specific marker genes. They showed that the proposed methods outperformed others in four different simulation scenarios. In addition, Celloscope was able to spatially distinguish between two subclasses of neurons in a real mouse brain ST data, and to delineates the spatial coordinates immune infiltration in human prostate tissues. Altogether, the results of this study are impressive. Still, some clarifications are needed and a few further questions should be addressed:*

***Comment 1:*** *First, the proposes method heavily depends on the choice of cell type specific markers. The manuscript provided the marker lists only for mouse brain and human prostate tissue, based on the spatial agreement of their expression across ST spots. However, there are other factors that impact the accuracy of ST deconvolution. One is the cell plasticity, which results in a continuum of immune phenotypes coexistence within the same system, complicating the distinction between "cell type" and "cell state". Another factor that many methods neglected is that immune cells can exhibit tumor/tissue-specific aberrant expression. It will be great if the authors can provide more details on marker selection and discuss how to address these issues with proposed method.*

**Response:** Thank you for your comments. Now we mention this matter in **Discussion (page 31-32)**, where we argue that Celloscope is readily applicable to localizing cell types in different states since marker genes could also define cell states.

Moreover, in this revision, we devised, implemented and described in detail a semi-automatic procedure for marker gene selection. This procedure filters out genes with low expression and selects marker genes based on their co-expression in spots, as quantified by the Kendall and Pearson correlation coefficients. The procedure is described in a new subsection **“Defining the marker genes for cell types”** in the main text, its source code in R is available in the GitHub repository and it was used to generate results in the manuscript **(Figures 3, 4, 5, 6)**.

***Comment 2:*** *Second, a dummy type was incorporate in the mode to account for cell type that exists in spot but is not represented by the cell type specific marker. However, the author didn't evaluate the algorithm's performance in scenarios when unknown cell types exist in ST spots.*

**Response:** Thank you for pointing this out. Actually, all data simulating scenarios assumed the presence of *the dummy type*, and the simulated spots contained unknown cell types, for which marker genes were not provided to the model **(Figure 2)**. Now we mention this fact explicitly in the section “**Celloscope’s results on simulated data prove exceptional performance of marker gene-based cell type deconvolution”**, page 4, under Figure 1.

***Comment 3:*** *Third, since the proposed method is marker-dependent without using any matched single-cell RNA-seq data, how is it different from other marker-dependent deconvolution methods developed for bulk transcriptomics data (non-spatial)? It will be interesting to see a comparison between Celloscope and methods for bulk transcriptomics data deconvolution, such as TIMER, EPIC, and quanTIseq.*

**Response: It is important to realize that the** aforementioned methods are designed for bulk RNA-seq data, which has much higher coverage, lower sparsity and variability than spatial transcriptomics data. Also, they are applied independently to each input bulk sample, and are thus not able to infer parameters taking into consideration all spots from ST without pooling them into a single pseudo bulk sample. Due to this fact one may expect issues when these data are applied to spatial transcriptomics.

Since the were designed to decompose immune cell types, we applied EPIC, quanTIseq, ABIS and xCell on human prostate data (section 3.1), where immune cell types were

expected to be present, running these methods both on each individual spot separately and on the pseudo bulk data. Only a few of these methods were able to run without errors. The results of the analyses, discussed in a new subsection ***Comparison to methods designed for immune cell type decomposition in bulk RNA-seq agree*** with the expectation. Indeed, they show that the approaches devised for bulk RNA-seq are unreliable for deconvoluting ST spots, and perform better on pseudo bulk, which completely lacks the spatial aspect **(Additional File 1: Figure S7 and Figure S8).**

***Comment 4:*** *Finally, the proposed methods, or any method benchmarked in this study, make explicit use of the spatial coordinates during modeling. The authors should at least discuss how the inference of cell types in a given spot should be influenced by the nearby spot.*

**Response:** Thank you for pointing it out! Indeed, our model is applied to each spot which is characterized by its spatial coordinate. However, apart from pre-selection of spots localized in areas of interest annotated by the pathologist, we do not model the influence between the nearby spots. In the **Discussion** of the revised manuscript, we now mention it as a possible extension of the model and future work, referring to two other studies, where information from neighboring spots was taken into consideration to solve tasks in analysis of spatial transcriptomics data but different than cell type deconvolution.

**Second round of review**

**Reviewer 1**

The authors have comprehensively addressed the previous round of comments.

One additional comment that may have been missed in the previous round: it is not clear if the full datasets used for the main results have been made publicly available. If not, the datasets should be made publicly accessible via some repository (e.g. processed R objects stored on Zenodo, or any other format and repository preferred by the authors), and/or links to the original data sources included in the "Availability of data and materials" section at the end of the manuscript.

**Reviewer 2**

In this revised manuscript, the authors performed substantial new analyses, significantly expanded the methodological description, and provided additional results and code. I have no further requests and I look forward to seeing this published.

**Authors Response**

**Point-by-point responses to the reviewers’ comments:**

Reviewer #1:

One additional comment that may have been missed in the previous round: it is not clear if the full datasets used for the main results have been made publicly available. If not, the datasets should be made publicly accessible via some repository (e.g. processed R objects stored on Zenodo, or any other format and repository preferred by the authors), and/or links to the original data sources included in the "Availability of data and materials" section at the end of the manuscript.

Datasets used for the main results had already been published. In the revised manuscript, we explicitly list data sources in the “Availability of data and materials”.

In the data directory of the repository we deposited data used to obtain the results in the paper: Mouse brain sagittal section data originally downloaded from https://support.10xgenomics.com/spatial-gene- expression/datasets/1.1.0/V1_Mouse_Brain_Sagittal_Anterior, as well as prior knowledge on marker genes as a binary matrix (matB.csv) and the results of cell counting procedure (n_cells.csv). Mouse brain coronal section data originally from https://support.10xgenomics.com/spatial-gene- expression/datasets/1.1.0/V1_Adult_Mouse_Brain) as well as prior knowledge on marker genes as a binary matrix (matB.csv) and the results of cell counting procedure (n_cells.csv). Gene expression for a subset of spots from Berglund et al [20] that were annotated as an inflammation by a pathologist, as well as prior knowledge on marker genes as a binary matrix (matB.csv) and the results of cell counting procedure (n_cells.csv). Simulated data is now deposited at the GitHub repository. All this information is now stated in the “Availability of data and materials” section in the revised manuscript.
